# Supplementary material for: Social determinants of the changing tuberculosis prevalence in Việt Nam: Analysis of population-level cross-sectional studies
Source: PLoS Med. 2022 Mar 18;19(3):e1003935. doi: 10.1371/journal.pmed.1003935 (PMC8932606; doi:10.1371/journal.pmed.1003935)
Supplement: S1 Text — (DOCX) [file pmed.1003935.s001.docx]

**S1 Text**

**Supporting information**

This document provides additional information related to the analysis reported in:

Foster et al. Social determinants of the changing tuberculosis prevalence in Việt Nam: analysis of population-level cross-sectional studies.

[1. Datasets 2](#_Toc86417536)

[2. Measuring socioeconomic position 2](#_Toc86417537)

[2.1. Relative measures 3](#_Toc86417538)

[2.2. Absolute wealth estimates 3](#_Toc86417539)

[3. Poverty indicators beyond the household 6](#_Toc86417540)

[4. Statistical analyses 8](#_Toc86417541)

[4.1. Variables 9](#_Toc86417542)

[4.2. Correlation 10](#_Toc86417543)

[4.3. Intra-level correlation 11](#_Toc86417544)

[4.4. Illness concentration curves and indices 15](#_Toc86417545)

[4.5. Statistical model selection 16](#_Toc86417546)

[4.5.1. Individual level 16](#_Toc86417547)

[4.5.2. Multi-level models 16](#_Toc86417548)

[4.5.3. Interaction terms (change over time) 17](#_Toc86417549)

[5. Sensitivity Analyses 17](#_Toc86417550)

[References 19](#_Toc86417551)

# Sampling

# The sampling strategy and selection of participants

# Figure A. Flow diagram of participant selection for the 1^st^ and 2^nd^ tuberculosis prevalence surveys in Việt Nam.

#
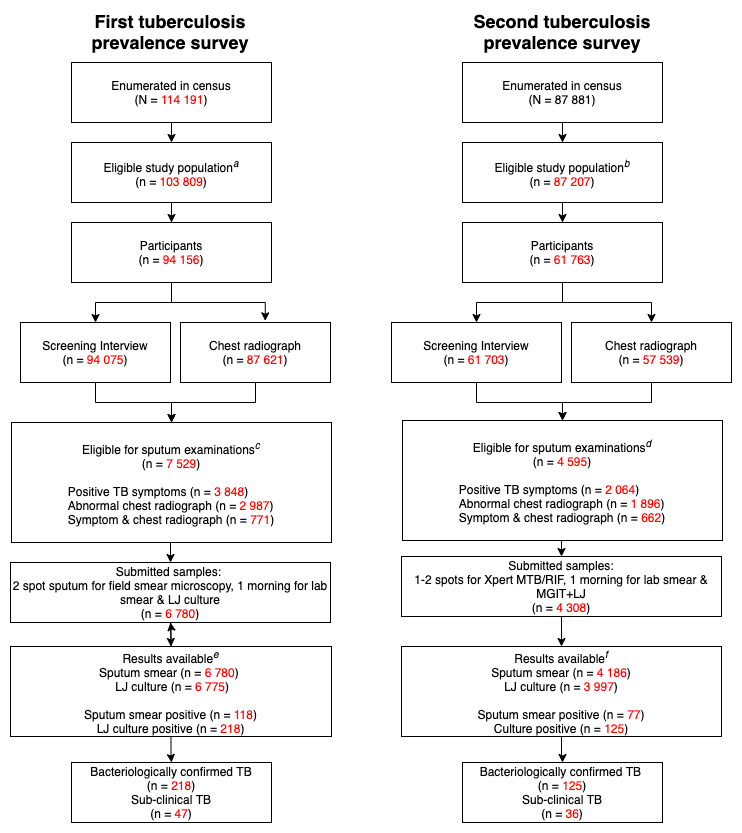


# In Figure A: Individuals who have lived in the household for 3months or more were enumerated in the first survey, compared to having lived in the household for two weeks or more in the second survey. Military camps and prisons were excluded. *^a, b^*The eligible study population included all who were mentally able to understand study procedures and risks and benefits. *^a^*In survey 1 individuals were excluded if they were not able to attend the survey field procedures. Participants were eligible for sputum examinations if they had a cough for 2 weeks or more with sputum if they were currently taking TB treatment or had been on TB treatment for 2 years or more preceding the survey, or if they had a chest x-ray suggesting tuberculosis.

# Datasets

In Table A, we show the proportion of items utilised by the participants interviewed in each of the TB prevalence surveys administered in 2007 and 2017. The items shown here represent household consumption questions asked during each of the surveys.

Table A. Proportion of individuals in each of the surveys reporting owning (1 = Yes) each of the household items in the 2007 and 2017 surveys respectively.

|  | **2007** | **2017** |
| --- | --- | --- |
| Clay Floor | 17% (14 204/ 83 614) | 13% (7 272/ 54 639) |
| Cooking with wood | 51% (42 250/ 83 615) | 37% (19 996/ 54 443) |
| Stereo system | 30% (11 217/ 37 766) | 30% (15 670/ 53 097) |
| Stereo cassette | 51% (45 668/ 89 165) | 44% (14 822/ 33 401) |
| Television | 81% (71 882/ 89 165) | 74% (24 825/ 33 401) |
| Motorbike | 71% (64 759/ 91 050) | 86% (49 370/ 57 343) |
| Car | 1% (1 126/ 83 587) | 3% (1 433/ 51 303) |
| Computer | 25% (9 279/ 37 607) | 24% (12 607/ 52 881) |
| Refrigerator | 79% (29 778/ 37 710) | 79% (41 992/ 52 994) |
| Washing machine | 48% (18 274/ 37 724) | 46% (24 232/ 53 034) |
| Water heater | 43% (16 283/ 37 630) | 39% (20 479/ 52 867) |
| Air conditioner | 28% (10 545/ 37 509) | 26% (13 804/ 52 742) |

# Measuring socioeconomic position

Krieger et al. 1997 defines socioeconomic position as a “concept that includes both resource-based and prestige-based measures…”. “Resource-based measures refer to material and social resources and assets, including income, wealth, educational credentials…”. “Prestige-based measures refer to individual’s rank or status in a social hierarchy, typically evaluated with reference to people’s access to and consumption of goods, services and knowledge as linked to their occupational prestige, income and education level” [1]. We use the term socioeconomic position rather than socioeconomic status to distinguish between different levels of resources as the primary unit of measurement, and prestige-related characteristics (societal class-based rules). Many of the consequences of these characteristics are geographically clustered. In this analysis, we include three measures of an individual’s socioeconomic position (1) household SEP estimated based on the consumption data collected during the prevalence survey, (2) provincial-level poverty, and (3) an absolute wealth estimate constructed based on the household’s SEP, the provincial gross domestic product (GDP) and the Gini coefficient.

These approaches to measuring poverty can be characterised as either relative or absolute. Relative approaches, such as household SEP measure households’ wealth relative to the wealth of other households in the sample. In constructing this measure, individuals of similar wealth are grouped together to compare differences in health or health seeking behaviour between groups of similar wealth. In contrast, absolute measures of wealth are measures wealth in absolute terms and then presented as people being poor if their income/wealth is below a certain agreed level. This includes the provincial level poverty measure and the absolute wealth measure. Approaches to estimate the absolute wealth of individuals are typically based on income, and therefore may not fully capture all the dynamics of poverty but are more easily comparable across time.

## Relative measures

Since household consumption questions were not the same between the two surveys and there were some missing values in the dataset related to some of the variables, we used the same consumption categories from 2007 in 2017 to generate the asset indices. This approach allows has been argued to improve comparability of the socioeconomic position measure between time points [2].  The limitations of a SEP measure based on consumption data is that it is vulnerable to changes in how representative the ownership of a particular item is of either wealth/ poverty. An item that may have been a symbol of wealth in 2007 may no longer be such in 2017. This especially relates to context specific changes in valuing telephones and vehicles. However, this approach makes comparing the wealth of TB patients in Việt Nam over time challenging. In addition to the relative measures of poverty (asset index), we estimated the absolute wealth (AWE) of each household so that we can estimate whether the resources available to households affected by TB changed between the two survey timepoints.

## Absolute wealth estimates

Following the approach proposed by Hruschka et al. 2015, we estimated an absolute wealth estimate for each of the households based on the consumption data and the households SEP, the per capita GDP in Việt Nam, and the Gini coefficient as a measure of dispersion [3].

STATA code

* setting data frame

set obs 10000

gen num_id = _n

lab var num_id "number observations"

* adding the GDP per capita as the average value

gen gdp_2007 = 906.284

lab var gdp_2007 "gdp per capita in 2007"

gen gdp_2017 = 2365.622

lab var gdp_2017 "gdp per capita in 2017"

* adding the gini coefficient as a measure of dispersion

gen gini = 35.7

Statistical distributions used to model wealth in populations include Pareto and log-normal distributions; we generated the following distributions:

**Normal distribution**

gen norm = rnormal(2365 35)

hist norm

**Pareto distribution**

egen double par = rndraw() , pareto(2365.62 35.7)

hist par

The Pareto distribution most approximate the wealth distribution in a country with a minimum wage and fairly equal distribution of wealth.

Table B. Quartile of household wealth.

| **quartile** | **mean** | **SD** | **min** | **max** |
| --- | --- | --- | --- | --- |
| 1 | 2374.99 | 5 | 2365.62 | 2380.72 |
| 2 | 2389.79 | 5.60 | 2380.73 | 2400.35 |
| 3 | 2412.55 | 7.72 | 2400.35 | 2427.07 |
| 4 | 2448.18 | 13.70 | 2427.07 | 2474.30 |

* Imputes the Absolute Wealth Estimate based on the households’ SEP (annual household wealth per capita)

* adding an absolute wealth estimate

gen awe_imp = .

replace awe_imp = 2374.99 if pca_revised_QUINT == 1

replace awe_imp = 2389.79 if pca_revised_QUINT == 2

replace awe_imp = 2412.55 if pca_revised_QUINT == 3

replace awe_imp = 2448.18 if pca_revised_QUINT == 4

sum awe_imp

A limitation of this approach is that the AWE is not adjusted for rural versus urban household incomes or what may be important in Việt Nam, is differences by geographical locations. The drivers of wealth in Việt Nam as identified in the Việt Nam Living Standards survey include the number of people in the household who are employed in non-agricultural settings and whether the household is in a urban or rural area [4].

# Poverty indicators beyond the household

To include community level impoverishment (as per our analytical framework) in our analysis, we combined the patient level data with community level socioeconomic indicators from publicly available data, from the World Bank [5,6].

Table C. Provincial socio-economic indicators.

|  | **Province name** | **Number of individuals in survey** | **Zone** | **Poverty: GSO-WB poverty headcount (%) 2009** | **MOLISA income-based poverty rate 2013** | **Percent of population living on less than $2/day** |
| --- | --- | --- | --- | --- | --- | --- |
| 1 | An Giang | 1 552 | South | 18.0 | 5.0 | 11.7 |
| 2 | Ba Ria - Vung Tau | 1 798 | South | 10.0 | 1.0 | 6.3 |
| 3 | Bac Giang | 3 152 | North | 24.0 | 10.4 | 16.7 |
| 4 | Bac Kan | 836 | North | 46.0 | 18.6 | 36.9 |
| 5 | Bac Lieu | 3 409 | South | 23.0 | 9.4 | 15.6 |
| 6 | Bac Ninh | 2 208 | North | 10.0 | 3.4 | 6.2 |
| 7 | Ben Tre | 1 742 | South | 10.0 | 8.6 | 5.8 |
| 8 | Binh Dinh | 2 177 | Centre | 17.0 | 9.9 | 10.5 |
| 9 | Binh Duong | 1 503 | South | 8.0 | 0.0 | 4.7 |
| 10 | Binh Phuoc | Not included |  | 17.0 | 4.5 | 11.3 |
| 11 | Binh Thuan | 542 | South | 21.0 | 4.9 | 14.7 |
| 12 | Ca Mau | 3 174 | South | 26.0 | 6.5 | 18.5 |
| 13 | Can Tho city | 3 166 | South/ Centre | 12.0 | 4.0 | 7.2 |
| 14 | Cao Bang | 2 257 | South | 53.0 | 24.2 | 45.4 |
| 15 | Da Nang city | 2 337 | Centre | 2.0 | 0.8 | 1.2 |
| 16 | Dak Lak | 3 464 | Centre | 30.0 | 12.3 | 24.3 |
| 17 | Dak Nong | 752 | Centre | 32.0 | 15.6 | 25.8 |
| 18 | Dien Bien | Not included |  | 71.0 | 35.2 | 65.1 |
| 19 | Dong Nai | 4 485 | South | 12.0 | 0.7 | 7.5 |
| 20 | Dong Thap | 4 610 | South/North | 16.0 | 7.5 | 9.5 |
| 21 | Gia Lai | 2 105 | Centre | 43.0 | 17.2 | 37.6 |
| 22 | Ha Giang | 2 120 | North | 71.0 | 27.0 | 64.8 |
| 23 | Ha Nam | Not included |  | 17.0 | 6.3 | 10.5 |
| 24 | Ha Noi | 8 968 | North | 5.0 | 1.0 | 2.9 |
| 25 | Ha Tinh | 2 549 | North/Centre | 22.0 | 10.7 | 13.8 |
| 26 | Hai Duong | 2 373 | North | 15.0 | 5.8 | 9.4 |
| 27 | Hai Phong city | 4 203 | North | 8.0 | 3.2 | 4.8 |
| 28 | Hau Giang | 1 926 | South | 20.0 | 11.6 | 12.9 |
| 29 | Ho Chi Minh city | 11 898 | South | 3.0 | 0.0 | 1.8 |
| 30 | Hoa Binh | 1 334 | North | 47.0 | 18.7 | 38.9 |
| 31 | Hung Yen | 2 305 | North | 13.0 | 5.1 | 8.0 |
| 32 | Khanh Hoa | 2 012 | Centre/ South | 16.0 | 4.3 | 10.9 |
| 33 | Kien Giang | 2 077 | South | 24.0 | 4.7 | 16.8 |
| 34 | Kon Tum | Not included |  | 48.0 | 19.2 | 42.6 |
| 35 | Lai Chau | 887 | North | 76.0 | 27.2 | 70.6 |
| 36 | Lam Dong | Not included |  | 22.0 | 4.1 | 16.9 |
| 37 | Lang Son | 2 230 | North | 46.0 | 18.0 | 36.8 |
| 38 | Lao Cai | 698 | North | 57.0 | 22.2 | 50.1 |
| 39 | Long An | 1 903 | South | 11.0 | 3.8 | 6.6 |
| 40 | Nam Dinh | 2 694 | North | 14.0 | 5.3 | 8.7 |
| 41 | Nghe An | 7 349 | North/Centre | 27.0 | 13.4 | 19.2 |
| 42 | Ninh Binh | 3 516 | North |  | 5.4 | 9.7 |
| 43 | Ninh Thuan | 2 039 | South | 35.0 | 9.3 | 26.7 |
| 44 | Phu Tho | 3 332 | North/Centre | 24.0 | 12.5 | 16.9 |
| 45 | Phu Yen | 910 | Centre | 22.0 | 13.0 | 14.9 |
| 46 | Quang Binh | 949 | Centre | 23.0 | 14.2 | 16.0 |
| 47 | Quang Nam | 3 256 | Centre | 23.0 | 14.9 | 16.4 |
| 48 | Quang Ngai | 2 509 | Centre | 24.0 | 14.9 | 17.1 |
| 49 | Quang Ninh | 2 149 | North | 12.0 | 2.4 | 8.4 |
| 50 | Quang Tri | Not included |  | 30.0 | 11.8 | 22.5 |
| 51 | Soc Trang | 2 098 | North/ South | 27.0 | 17.0 | 19.6 |
| 52 | Son La | 2 018 | North | 64.0 | 27.0 | 55.7 |
| 53 | Tay Ninh | 3 163 | South | 12.0 | 1.7 | 7.1 |
| 54 | Thai Binh | 3 084 | North | 19.0 | 4.6 | 12.3 |
| 55 | Thai Nguyen | 3 396 | North | 22.0 | 11.6 | 15.4 |
| 56 | Thanh Hoa | 5 503 | North | 26.0 | 13.1 | 18.8 |
| 57 | Thua Thien Hue | 759 | Centre | 19.0 | 6.4 | 13.1 |
| 58 | Tien Giang | 1 609 | South | 10.0 | 6.3 | 5.5 |
| 59 | Tra Vinh | 2 059 | South | 22.0 | 14.0 | 15.5 |
| 60 | Tuyen Quang | 2 822 | North | 40.0 | 17.9 | 31.4 |
| 61 | Vinh Long | 2 109 | South | 12.0 | 4.6 | 7.2 |
| 62 | Vinh Phuc | Not included |  | 12.0 | 4.9 | 7.4 |
| 63 | Yen Bai | 2 452 | North | 45.0 | 25.4 | 37.5 |

Data source: [mapVIETNAM](http://www5.worldbank.org/mapvietnam/) .

1. **Statistical analyses**

To account for within-cluster variation and time-dependent change between 2007 and 2017, we followed the following steps for checking the assumptions associated with statistical models: (1) We used the Hausman test to check if random error is correlated with individual effects. The Hausman test can be used to assess whether a random- or fixed effects model should be used. (2) To test for group/ cross-sectional effects, we ran a LSDV model against a pooled regression and checked whether there was a significant improvement in the rsquare. (3) Checked for autocorrelation in the model using xtgls to assess correlation across the cross-sections, ar(1) with panels and heteroskedasticity and no cross-panel correlation and xtgee for richer designation of within panel correlation as long as correlation applies to all panels.

## Variables

The variables included in the analyses and shown in Figure 1 of the manuscript are detailed in Table D below.

Table D. Description of variables included in the analysis.

| **Variable name** | **Variable label** | **Data type** | **Description** |
| --- | --- | --- | --- |
| Individual |  |  |  |
| Tbcase | TB | Y/N | microbiologically confirmed tuberculosis. |
| sub_tbcase | Subclinical TB | Y/N | sub-clinical tuberculosis |
| Survey | Timepoint | 2007/ 2017 |  |
| age_cat | Age categories | 15-24  25-34  35-44  45-54  55-64  > 65 |  |
|  |  |  |  |
| sex_final | Gender | F/M | Gender |
| symptoms_any | Symptoms | Y/N | Presence of TB-associated symptoms. |
| Past_TB_care | Past TB | Y/N | TB treatment in the past. |
| Household |  |  |  |
| HHH | HHH gender | F/M | Gender of the self-declared head of the household. |
| quartile | SEP | 1 lowest  2 lower middle  3 upper middle  4 highest | Household socioeconomic position based on consumption data. |
| Region | Geographical | North/ Centre/ South |  |
| stratum_id | Residence | Urban/ Rural/ Remote | Type of residence |
| wood_cook_F1 | Cooking fuel | Y/N | Cooking on wood |
| awe | AWE | continuous | Household absolute wealth estimate |
| District |  |  |  |
| District poverty consumption |  | continuous | District poverty headcount % (2009) |
| District poverty income |  | continuous | District MOLISA income-based poverty rate (2013) |

## Correlation

Correlogram showing the correlation between the indicators included in the analysis and tuberculosis disease. Pearson’s correlations matrix.

Pwcorr tbcase survey age_final sex_final ethnic_F1 symptoms_any quartile_Nicola_All stratum_id wood_cook_F1

| tbcase survey age_fi~l sex_fi~l ethnic~1 sympto~y quarti~l

-------------+---------------------------------------------------------------

tbcase | 1.0000

survey | -0.0032 1.0000

age_final | 0.0291 0.1796 1.0000

sex_final | 0.0303 -0.0126 -0.0506 1.0000

ethnic_F1 | -0.0012 0.0288 -0.0089 -0.0266 1.0000

symptoms_any | -0.0124 -0.0296 0.0673 0.0256 -0.0434 1.0000

quartile_N~l | -0.0030 -0.0784 -0.0664 -0.0392 0.3429 -0.0250 1.0000

stratum_id | -0.0014 -0.0053 0.0191 0.0020 -0.0092 0.0302 0.0759

wood_cook_F1 | -0.0041 -0.0703 -0.0637 -0.0611 0.6802 -0.0345 0.4262

| stratu~d wood_c~1

-------------+------------------

stratum_id | 1.0000

wood_cook_F1 | 0.0392 1.0000

pwcorr tbstat age_final sex_final symptoms_any ethnic_F1 pca_revised_QUINT survey province pastb_care_F5, sig star(0.5)

. pwcorr tbstat age_final sex_final symptoms_any ethnic_F1 quartile_Nicola_All province pastb_care_F5, sig star(0.5)

(province ignored because string variable)

| tbstat age_fi~l sex_fi~l sympto~y ethnic~1 quarti~l pastb_~5

-------------+---------------------------------------------------------------

tbstat | 1.0000

|

|

age_final | 0.0368* 1.0000

| 0.0000

|

sex_final | 0.0387* -0.0506* 1.0000

| 0.0000 0.0000

|

symptoms_any | -0.0158* 0.0673* 0.0256* 1.0000

| 0.0000 0.0000 0.0000

|

ethnic_F1 | 0.0017 -0.0089* -0.0266* -0.0434* 1.0000

| 0.5413 0.0019 0.0000 0.0000

|

quartile_N~l | -0.0025* -0.0664* -0.0392* -0.0250* 0.3429* 1.0000

| 0.3371 0.0000 0.0000 0.0000 0.0000

|

pastb_care~5 | -0.0030 0.0157* 0.0454* 0.0091* -0.0043 0.0063* 1.0000

| 0.7405 0.0844 0.0000 0.3209 0.6740 0.4957 |

## Intra-level correlation

Correlation between variables that are on separate levels (individual, household and province-level variables) are assessed using the Hausmann test.

The cluster adjusts the standard errors for variation within clusters.

. logit tbstat age_final i.sex_final i.ethnic_F1 symptoms_any, vce(cluster province)

Iteration 0: log pseudolikelihood = -947.49249

Iteration 1: log pseudolikelihood = -912.0981

Iteration 2: log pseudolikelihood = -906.53023

Iteration 3: log pseudolikelihood = -906.49139

Iteration 4: log pseudolikelihood = -906.49136

Logistic regression Number of obs = 32,907

Wald chi2(4) = 55.37

Prob > chi2 = 0.0000

Log pseudolikelihood = -906.49136 Pseudo R2 = 0.0433

(Std. Err. adjusted for 48 clusters in province)

------------------------------------------------------------------------------

| Robust

tbstat | Coef. Std. Err. z P>|z| [95% Conf. Interval]

-------------+----------------------------------------------------------------

age_final | .0306962 .0066885 4.59 0.000 .0175869 .0438055

|

sex_final |

male | 1.319406 .2638722 5.00 0.000 .8022257 1.836586

|

ethnic_F1 |

Kinh | -.2241511 .4005156 -0.56 0.576 -1.009147 .5608451

symptoms_any | -1.052445 .5235994 -2.01 0.044 -2.078681 -.0262094

_cons | -7.677256 .5859589 -13.10 0.000 -8.825714 -6.528798

logit tbstat age_final i.sex_final i.ethnic_F1 symptoms_any i.survey, vce(cluster province)

note: 0.survey omitted because of collinearity

Iteration 0: log pseudolikelihood = -947.49249

Iteration 1: log pseudolikelihood = -912.0981

Iteration 2: log pseudolikelihood = -906.53023

Iteration 3: log pseudolikelihood = -906.49139

Iteration 4: log pseudolikelihood = -906.49136

Logistic regression Number of obs = 32,907

Wald chi2(4) = 55.37

Prob > chi2 = 0.0000

Log pseudolikelihood = -906.49136 Pseudo R2 = 0.0433

(Std. Err. adjusted for 48 clusters in province)

------------------------------------------------------------------------------

| Robust

tbstat | Coef. Std. Err. z P>|z| [95% Conf. Interval]

-------------+----------------------------------------------------------------

age_final | .0306962 .0066885 4.59 0.000 .0175869 .0438055

|

sex_final |

male | 1.319406 .2638722 5.00 0.000 .8022257 1.836586

|

ethnic_F1 |

Kinh | -.2241511 .4005156 -0.56 0.576 -1.009147 .5608451

symptoms_any | -1.052445 .5235994 -2.01 0.044 -2.078681 -.0262094

|

survey |

1st survey | 0 (omitted)

_cons | -7.677256 .5859589 -13.10 0.000 -8.825714 -6.528798

xtreg tbstat age_final i.sex_final i.ethnic_F1 symptoms_any i.stratum_id i.pca_revised_QUINT, re vce(cluster province)

Approaches for assessing the relationships between clusters and SES (a) Constructing a neighbourhood deprivation index [7] or (b) Hierarchical clustering to group similar neighbourhoods together [8].

**Studying residential socioeconomic factors**

**Area level characteristics, hierarchical clustering**

[**https://www.sciencedirect.com/topics/computer-science/hierarchical-cluster-analysis**](https://www.sciencedirect.com/topics/computer-science/hierarchical-cluster-analysis)

Neighbourhood SES for health inequalities analysis

https://pubmed.ncbi.nlm.nih.gov/23537275/

. xtlogit tbstat age_final sex_final symptoms_any ethnic_F1 pca_revised_QUINT survey province_CAT, fe

note: survey omitted because of collinearity

note: multiple positive outcomes within groups encountered.

Iteration 0: log likelihood = -832.1861

Iteration 1: log likelihood = -832.1861

Conditional fixed-effects logistic regression Number of obs = 28,743

Group variable: survey Number of groups = 1

Obs per group:

min = 28,743

avg = 28,743.0

max = 28,743

LR chi2(6) = 67.07

Log likelihood = -832.1861 Prob > chi2 = 0.0000

-----------------------------------------------------------------------------------

tbstat | Coef. Std. Err. z P>|z| [95% Conf. Interval]

------------------+----------------------------------------------------------------

age_final | .0278761 .0055609 5.01 0.000 .0169769 .0387753

sex_final | 1.214838 .2458857 4.94 0.000 .7329106 1.696765

symptoms_any | -.9850941 .315418 -3.12 0.002 -1.603302 -.3668863

ethnic_F1 | -.0371839 .2509319 -0.15 0.882 -.5290013 .4546334

pca_revised_QUINT | -.2202529 .0807601 -2.73 0.006 -.3785398 -.0619659

survey | 0 (omitted)

province_CAT | -.0009056 .0053822 -0.17 0.866 -.0114545 .0096434

-----------------------------------------------------------------------------------

. estimates store fixed

. hausman random fixed

---- Coefficients ----

| (b) (B) (b-B) sqrt(diag(V_b-V_B))

| random fixed Difference S.E.

-------------+----------------------------------------------------------------

age_final | .0278777 .0278761 1.55e-06 .0000473

sex_final | 1.214886 1.214838 .0000484 .0013796

symptoms_any | -.9851309 -.9850941 -.0000367 .0015589

ethnic_F1 | -.0371854 -.0371839 -1.50e-06 .0019047

pca_revise~T | -.220264 -.2202529 -.0000112 .0005726

province_CAT | -.0009056 -.0009056 -6.51e-08 .0000396

------------------------------------------------------------------------------

b = consistent under Ho and Ha; obtained from xtlogit

B = inconsistent under Ha, efficient under Ho; obtained from xtlogit

Test: Ho: difference in coefficients not systematic

chi2(6) = (b-B)'[(V_b-V_B)^(-1)](b-B)

= 0.00

Prob>chi2 = 1.0000

The Hausman test detects endogenous regressors in a regression model – values that are determined by other variables in the system. In the panel data analysis, the Hausman test will help you choose between fixed effects models or random effects models. The Null hypothesis is that the model is fixed effects and essentially the test looks to see whether there is a correlation between unique errors and the regressors in the model.

## Illness concentration curves and indices

Standard versus generalised concentration indices [9,10].

The equation for the concentration index (C) can formally be expressed as:

|  | $C= 2/\mu cov(h\_i,r)$ | ………..(1) |
| --- | --- | --- |

Where h_i_ is the health sector variable (here, TB disease) for individual *i*, $\mu$ is the mean of the health sector variable, and r_i_ is the fractional rank of individual *i* in the assets distribution. The index is bounded between -1 and 1 where, if the health sector variable represents disease as in this case, a negative value of the concentration index means that ill health (TB disease) is concentrated among the poor. C depends only on the relationship between the health variable and the rank of the asset variable.

Estimating the confidence interval for the combined sample:

. conindex tbstat, rankvar(quartile_Nicola_All) truezero robust compare(survey)

------------------------------------------------------------------------------+

Index: | No. of obs. | Index value | Robust std. error | p-value |

-------------------+-------------+-------------+-------------------+----------|

CI | 147235 | -.02298591 |.02536095 | 0.3648 |

------------------------------------------------------------------------------+

For groups:

CI for group 1: survey = 0

------------------------------------------------------------------------------+

Index: | No. of obs. | Index value | Robust std. error | p-value |

-------------------+-------------+-------------+-------------------+----------|

CI | 90975 | -.09297746 |.03311852 | 0.0050 |

------------------------------------------------------------------------------+

CI for group 2: survey = 1

------------------------------------------------------------------------------+

Index: | No. of obs. | Index value | Robust std. error | p-value |

-------------------+-------------+-------------+-------------------+----------|

CI | 56260 | .07679663 |.0399233 | 0.0544 |

------------------------------------------------------------------------------+

Test for the statistical differences between the two concentration curves.

Test for stat. significant differences with Ho: diff=0 (assuming equal variances)

---------------------------------------+

F-stat = 10.292039 | p-value= 0.0013 |

---------------------------------------+

Test for stat. significant differences with Ho: diff=0 (large sample assumed)

--------------------------------------------------------------------------------+

Diff. = .16977409 | Std. err. = .05187202 | z-stat = 3.27| p-value = 0.0011|

--------------------------------------------------------------------------------+

## Statistical model selection

For each of the models, the Akaike Information Criterion (AIC) was estimated and compared between models as an approach for assisting in evaluating how well different models fit the data. The relationship between the dependent variables and independent variables were drawn as a causal model which is shown in Figure 1. In evaluating the AIC, lower scores and fewer parameters are better.

The equation for AIC is expressed as:

|  | $AIC=2K-2ln(L)$ | (1) |
| --- | --- | --- |

where K is the number of independent variables and L is the log-likelihood estimate.

### Individual level

Assess the increase in log odds per group using a logistic regression/ *X^2^* test for trend.

### Multi-level models

Log binomial mixed effects multi-level models with group- and individual-level intercepts were used to explain the social determinants of the change in TB prevalence over time, while taking into account the clustered nature of the data which has individuals nested in districts within households. Three-level regression models were used to investigate relationships between individual-, household- and provincial-level effects. We used the log binomial rather than logit models, because logit models tend to overestimate the prevalence ratio, though in rare events risk ratio approximate odds ratio in logit models. The model was specified as follows:

The equation for the multi-level model is expressed as:

$$\log\left( \frac{\hat{\pi}}{1-\hat{\pi}} \right)= \beta_{0}+ \beta_{1}x_{1}+\cdots+ \beta_{p}x_{p}$$

$$Y \sim Binomial(\hat{\pi})$$

Where Y has a binomial distribution with parameter $\pi$, and where $\hat{\pi}$ is the predicted probability that Y = 1.

### Interaction terms (change over time)

When the analysis moves towards assessing the change in TB prevalence over time, and repeated measures, I distinguish between the (1) between-subject effect and (2) the within-subject factor. The between-subject effect is “TB case” and its error term is the individual nested in TB case (i| TB case). The within-subject factor is time included as the survey. Its error term is the residual error for the model. The repeated measures assumption is that the within-subject covariance structure is exchangeable and therefore variances at time intervals are expected to be equal and covariances are expected to be equal to one another.

# Sensitivity Analyses

In a sensitivity analysis, we considered whether the results of the analyses hold if we focus on subclinical TB. For the purposes of our analysis, subclinical TB is defined as a patient who has bacteriologically confirmed tuberculosis but no TB-associated symptoms other than a cough.

Table E. Sensitivity analyses – subclinical tuberculosis.

|  | **2007 survey** |  | **2017 survey** |  |
| --- | --- | --- | --- | --- |
| **TB prevalence** | **β (95% CI)** | **pvalue** | **β (95% CI)** | **pvalue** |
| Age |  |  |  |  |
| 25-34 | 0.083 (-1.99; 2.17) | 0.937 | -0.891 (-2.12; 0.34) | 0.158 |
| 35-44 | 0.309 (-1.70; 2.32) | 0.763 | -0.607 (-1.42; 0.21) | 0.145 |
| 45-54 | 0.805 (-1.19; 2.79) | 0.429 | -0.820 (-1.58; -0.05) | 0.036 |
| 55-64 | 0.930 (-1.08; 2.94) | 0.365 | 0.176 (-0.44; 0.79) | 0.576 |
| >65 | 1.341 (-0.65; 3.33) | 0.187 | - |  |
| Gender |  |  |  |  |
| Female | Ref |  | Ref |  |
| Male | 1.08 (0.58; 1.58) | 0.000 | 1.17 (0.52; 1.83) | 0.000 |
| Region |  |  |  |  |
| North | Ref |  | Ref |  |
| Centre | -0.14 (-0.70; 0.40) | 0.598 | 0.28 (-0.41; 0.99) | 0.42 |
| South | 0.120 (-0.29; 0.53) | 0.563 | 0.61 (0.02; 1.19) | 0.04 |
| Type of residence |  |  |  |  |
| Urban | Ref |  | Ref |  |
| Rural | 1.073 (0.781; 1.474) | 0.663 | -0.484 (-1.06; 0.09) | 0.100 |
| Remote | 0.734 (0.511; 1.055) | 0.094 | 0.605 (0.02; 1.19) | 0.042 |
| Household socioeconomic position |  |  |  |  |
| Lowest | Ref |  | Ref |  |
| Lower Middle | 0.259 (-0.202; 0.719) | 0.271 | 0.023 (-0.984; 1.029) | 0.965 |
| Upper Middle | -0.016 (-0.698; 0.666) | 0.963 | 0.199 (-0.702; 1.100) | 0.664 |
| Highest | -0.442 (-1.015; 0.132) | 0.131 | 0.052 (-0.942; 1.046) | 0.919 |
| AIC | 0.052 |  | 0.039 |  |

# References

1. Krieger N, Williams D, Moss N. Measuring social class in US public health research: concepts, methodologies and guidelines. Annu Rev Public Health. [cited 19 Apr 2021]. doi:10.1146/annurev.publhealth.18.1.341

2. Ataguba JE, Akazili J, McIntyre D. Socioeconomic-related health inequality in South Africa: evidence from General Household Surveys. Int J Equity Health. 2011;10: 48. doi:10.1186/1475-9276-10-48

3. Hruschka DJ, Gerkey D, Hadley C. Estimating the absolute wealth of households. Bull World Health Organ. 2015;93: 483–490. doi:10.2471/BLT.14.147082

4. General Statistics Office. The VIet Nam Household Living Standards Survey 2014. Available: https://www.gso.gov.vn/en/data-and-statistics/2019/03/result-of-the-vietnam-household-living-standards-survey-2014/

5. The World Bank Data: Viet Nam. Available: https://data.worldbank.org/country/VN

6. The World Bank. The World Bank mapVIETNAM dataset. Available: http://www5.worldbank.org/mapvietnam/

7. Harling G, Ehrlich R, Myer L. The social epidemiology of tuberculosis in South Africa: a multilevel analysis. Soc Sci Med 1982. 2008;66: 492–505. doi:10.1016/j.socscimed.2007.08.026

8. Mirowsky JE, Devlin RB, Diaz-Sanchez D, Cascio W, Grabich SC, Haynes C, et al. A novel approach for measuring residential socioeconomic factors associated with cardiovascular and metabolic health. J Expo Sci Environ Epidemiol. 2017;27: 281–289. doi:10.1038/jes.2016.53

9. O’Donnell O, O’Neill S, Van Ourti T, Walsh B. conindex: Estimation of concentration indices. Stata J. 2016;16: 112–138.

10. World Bank, editor. Chapter 8: Concentration Index. Analyzing health equity using household survey data. Available: https://www.worldbank.org/en/topic/health/publication/analyzing-health-equity-using-household-survey-data
