# Supplementary material for: Social determinants of the changing tuberculosis prevalence in Việt Nam: Analysis of population-level cross-sectional studies
Source: PLoS Med. 2022 Mar 18;19(3):e1003935. doi: 10.1371/journal.pmed.1003935 (PMC8932606; doi:10.1371/journal.pmed.1003935)
Supplement: Attachment — Submitted filename: PMEDICINE-D-21-03248_reviewed_SN.pdf [file pmed.1003935.s003.pdf]

PLOS Medicine

Reducing the burden of tuberculosis among the poor: social determinants of the changing tuberculosis prevalence in Viet Nam

--Manuscript Draft--

**Manuscript Number:**	PMEDICINE-D-21-03248
**Full Title:**	Reducing the burden of tuberculosis among the poor: social determinants of the changing tuberculosis prevalence in Viet Nam
**Short Title:**	Social determinants of the changing tuberculosis prevalence in Viet Nam
**Article Type:**	Research Article
**Section/Category:**	Tuberculosis
**Abstract:**	Background<br>An ecological relationship between economic development and reduction in tuberculosis prevalence has been observed. Between 2007 and 2017, Viet Nam experienced rapid economic development with equitable distribution of resources and a 37% reduction in TB prevalence. Analysing consecutive TB prevalence surveys, we examined how the reduction in TB prevalence was concentrated between socio-economic groups.<br><br>Methods<br>We combined data from two nationally representative Viet Nam TB prevalence surveys to district-level measures of poverty. We constructed asset indices using principal component analysis of consumption data. Illness concentration indices were estimated to measure socio-economic position inequality in TB prevalence. We fitted multi-level models to investigate relationships between change in TB prevalence, individual risks, household SEP and neighbourhood poverty.<br><br>Findings<br>Data from 94156 (2007) and 61763 (2017) individuals were included. Of people with microbiologically confirmed TB, 21.6%(47/218) in 2007 and 29.0%(36/124) in 2017 had subclinical disease. The illness concentration index changed from -0·10(95%CI: -0·08, -0·16, p-value=0·003) in 2007 to 0·07(95%CI: 0·06, 0·18, p-value=0·158) in 2017, indicating that TB was concentrated among the poorest households in 2007, with a shift towards more equal distribution between rich and poor in 2017. These findings were similar for subclinical TB. After controlling for provincial poverty levels, we found that the significance of the reduction in TB prevalence reduced suggesting that changes in neighbourhood poverty contributed to the explanation.<br><br>Interpretation<br>We found that with equitable economic growth and a reduction in TB burden, TB became less concentrated among the poor in Viet Nam.<br><br>Funding<br>European Research Council
**Additional Information:**	
**Question**	**Response**
**Financial Disclosure**<br><br>Enter a financial disclosure statement that describes the sources of funding for the work included in this submission. Review the submission guidelines for detailed requirements. View published research articles from *PLOS Medicine* for specific examples.	This work was supported by a European Research Council starting grant (TBornotTB, action number 757699). The funder had no involvement in the study design; in the collection, analysis and interpretation of data; in the writing of the report; and in the decision to submit the paper for publication.

Powered by Editorial Manager® and ProduXion Manager® from Aries Systems Corporation

This statement is required for submission and **will appear in the published article** if the submission is accepted. Please make sure it is accurate.<br><br>**Unfunded studies**<br>Enter: *The author(s) received no specific funding for this work.*<br><br>**Funded studies**<br>Enter a statement with the following details:<br>• Initials of the authors who received each award<br>• Grant numbers awarded to each author<br>• The full name of each funder<br>• URL of each funder website<br>• Did the sponsors or funders play any role in the study design, data collection and analysis, decision to publish, or preparation of the manuscript?<br>• **NO** - Include this sentence at the end of your statement: *The funders had no role in study design, data collection and analysis, decision to publish, or preparation of the manuscript.*<br>• **YES** - Specify the role(s) played.<br><br>* typeset	
**Competing Interests**<br><br>Use the instructions below to enter a competing interest statement for this submission. On behalf of all authors, disclose any competing interests that could be perceived to bias this work—acknowledging all financial support and any other relevant financial or non-financial competing interests.<br><br>This statement **will appear in the published article** if the submission is accepted. Please make sure it is accurate. View published research articles from *PLOS Medicine* for specific examples.	The authors have declared that no competing interests exist.

**NO authors have competing interests**<br><br>Enter: *The authors have declared that no competing interests exist.*<br><br>**Authors with competing interests**<br>Enter competing interest details beginning with this statement:<br><br>*I have read the journal's policy and the authors of this manuscript have the following competing interests: [insert competing interests here]*<br><br>* typeset<br><br>This statement is required for submission and **will appear in the published article** if the submission is accepted. Please make sure it is accurate and that any funding sources listed in your Funding Information later in the submission form are also declared in your Financial Disclosure statement.	

Powered by Editorial Manager® and ProduXion Manager® from Aries Systems Corporation

Main Text Click here to access/download;Manuscript;manuscript.pdf

Reducing the burden of tuberculosis among the poor: social determinants of the changing tuberculosis prevalence in Viet Nam

Nicola Foster, PhD[1*],
Hai V Nguyen, MD[2,3],
Prof Nhung V Nguyen, PhD[2],
Prof Hoa B Nguyen, PhD[2],
Edine W Tiemersma, PhD[4],
Prof Frank GJ Cobelens, PhD[3],
Matthew Quaife, PhD[1],
Rein MGJ Houben, PhD[1]

[1]Department of Infectious Disease Epidemiology, London School of Hygiene and Tropical Medicine, Keppel Street, London, WC1E 7HT, United Kingdom
[2]Viet Nam National Tuberculosis Programme, 463 Hoang Hoa Tham, Hanoi, Viet Nam
[3]Department of Global Health and Amsterdam Institute of Global Health and Development, Amsterdam University Medical Centres, AHTC building, Paasheuvelweg 25, 1105 BP Amsterdam, the Netherlands
[4]KNCV Tuberculosis Foundation, Maanweg 174, 2516 AB Den Haag, the Netherlands

Word count: Abstract 249/ 250; Text 3 345 / 3 500
References: 29/ 30
Figures: 5; **Tables:** 3

Corresponding author:
*Nicola Foster
Department of Infectious Disease Epidemiology
Faculty of Epidemiology and Public Health, London School of Hygiene and Tropical Medicine,
London WC1E 7HT, UNITED KINGDOM,
e-mail address: nicola.foster@lshtm.ac.uk

Data sharing statement: The minimal dataset required to replicate this analysis and data dictionary is available from LSHTM Data Compass [LINK TO BE PROVIDED]. Individual-level data are available to researchers who meet the criteria for access to confidential data upon request to the Institutional Review Board of Vietnam National Lung Hospital (bvptw@bvptw.org).

Funding: This work was supported by a European Research Council starting grant (TBornotTB, action number 757699). The funder had no involvement in the study design; in the collection, analysis and interpretation of data; in the writing of the report; and in the decision to submit the paper for publication.

Key words: tuberculosis; social determinants of health; prevalence studies

Research in context

Evidence before this study

Historically, large reductions in tuberculosis prevalence globally have been ascribed to changes in living standards, such as housing and nutrition, that come with economic development. Previous studies have shown that social protection policies (a component of economic development) may reduce tuberculosis incidence, but that these gains are dependent on amount invested in social protection policies. However, direct evidence of the interaction between economic growth and TB burden is limited and missing with regard to equity.

Added value of this study

We used data from consecutive TB prevalence surveys conducted during a time of rapid economic growth in Viet Nam to analyse the associations between equitable economic development and reductions in TB prevalence. We found a significant shift in the distribution of TB from disproportionately affecting the poor towards a more equitable distribution of the reduced TB prevalence among the population, closely linked to neighbourhood poverty indicators.

Implications of all the available evidence

Our work contributes to the body of evidence of social determinants of TB prevalence. Furthermore, the work shows a more equitable burden of TB disease is possible in the context of rapid, and equitable, economic growth. Further work is required to understand how improvements in healthcare services contribute to or mediate the drive towards a more equitable burden of tuberculosis.

Introduction

The relationship between bacteriologically confirmed tuberculosis disease (TB) and poor socio-economic position (SEP) is well documented. Ecological studies have attributed sustained reductions in TB prevalence to improvements in housing, air quality and nutrition that accompany economic development and social policy such as social protection (1). The World Health Organisation's End TB strategy and the UN's Sustainable Development Goal 3 recognise the importance of the control and elimination of communicable diseases associated with poverty, as an outcome and step towards economic development.

Recent empirical work have attempted to quantify the effect of social protection on programmatic indicators, such as prevalence and case detection rates (1–8). Carter et al. considered a component of economic development policy, social protection, and how policy may affect TB incidence (2). Social protection refers to policies designed to reduce poverty through improvements in the labour market, and support for the poor and the sick. They found that social protection may reduce the incidence of TB by 76% (2). In evaluating the relationship between social protection and economic development, Siroka et al. found that tuberculosis prevalence is reduced with increased spending on social protection though this effect plateaued when spending more than 11% of gross domestic product (GDP) on social protection (5). Although these studies provide evidence that economic growth and social protection are associated with reductions in TB burden, they did not explore how the distribution of TB prevalence changes during economic growth.

Viet Nam is an example of a country that has experienced notable sustained economic growth. National TB prevalence surveys were conducted in Viet Nam in 2007 and 2017 (9,10). When differences in TB screening and diagnostic practices were accounted for, a comparative study showed a decline in TB prevalence over the ten-year period (11). The study found a 37% reduction in the prevalence of culture-positive TB, a 53% reduction in the prevalence of smear-positive TB, and no significant reduction in smear-negative - or subclinical TB. The change in TB prevalence was more pronounced among men, people living in rural areas, as well as in provinces in the North and South of the country (11).

In 1986 a series of economic reforms, the Dổi Mới Policy, were introduced which included investments in health and education (12). Since then, Viet Nam has experienced rapid and sustained economic growth, with GDP per capita rising from $230 in 1985 to $906 in 2007 and $2 343 in 2017. During this period, income inequality as measured by the Gini coefficient has remained stable for over a decade (35.8 in 2006 and 35.7 in 2018) (13,14). The increase in GDP per capita with an unchanging Gini coefficient suggests that the benefits of rapid economic development observed in Viet Nam have been

distributed equitably among the population, an example of shared prosperity. In the analysis presented here, we use the opportunity of measured longitudinal trends in both poverty and TB burden to examine the individual, household and neighbourhood social determinants of the reduction in TB prevalence in Viet Nam.

Methods

We combined individual-level data from two cross-sectional tuberculosis prevalence surveys to measure the social determinants of changes in TB prevalenc[illegible]he socioeconomic position (SEP) of households were estimated by constructing indices from consumption data and an illness concentration curve to represent the distribution of illness. Associations between TB prevalence, individual risk factors and household SEP within neighbourhoods were estimated by fitting mixed-effects multilevel models (15–17).

Causal pathways

Figure 1 shows the causal model for the analysis arranged by individual risks, household- and neighbourhood effects (18). Causal models are representations of assumed causal structures and provide a framework for discussing study design, variables included and how this may affect our understanding of the measure of interest (19).

Individual risk of developing TB is influenced by age, gender and previous treatment and how these intersect with household risk and household economic position in the neighbourhood (20). Transmission of tuberculosis is spatially concentrated in neighbourhoods (8). Similarly, economic development leads to increased opportunities in neighbourhoods, and depending on how wealth is distributed, there may be a reduction in unemployment, greater assistance to households in need and therefore more resources per capita. Equitable economic development improves neighbourhood economy which improves living conditions (reduced crowding) and reduces the period of transmission in neighbourhoods. Furthermore, improvements in the neighbourhood economy will increase household resources, reducing malnutrition and improving households' ability to seek healthcare. Comparatively wealthier households will have greater ability to negotiate access to neighbourhood resources such as housing and health services therefore lowering their risk of tuberculosis. If symptomatic (clinical tuberculosis), individuals would be more likely to seek and receive TB care, reducing transmission periods. However, if not symptomatic (subclinical), diagnosis within health services focused on passive TB case finding may be delayed until onset of clinical disease, leading to increased TB prevalence in the population (21,22).

[INSERT FIGURE 1]

Viet Nam national TB prevalence surveys and case definitions

Nationally representative Viet Nam TB prevalence surveys were conducted in 60 of 63 provinces in 2007 and 2017 (9,10). Households were identified for inclusion in the surveys by multistage sampling whereby first districts and then communes were selected proportional to population size. Sub-communes were selected by random sampling and all households in selected sub-communes were included. Individuals from selected households were eligible if they were older than 15. Screening procedures included questions on cough and treatment history followed by chest radiography, sputum smear microscopy and solid Löwenstein-Jensen (LJ) culture. Individuals reporting a cough for at least 2 weeks, haemoptysis, previous TB treatment, or with an abnormal chest X-ray were considered screen positive. In the first survey 8.0% (7 529/ 94 156) screened positive compared to 7.4% (4 595/ 61 763) in the second survey (23).

There were improvements in diagnostic technology between the two surveys. For comparability, an individual was considered to have bacteriologically confirmed TB if they were screen-positive, had a smear microscopy test and at least one positive LJ culture. Individuals were considered to have subclinical TB if they had not reported any symptoms but had at least one positive LJ culture.

Data from the prevalence surveys were matched to provincial level measures of poverty using data from the World Bank, the percentage of people living on less than $2 per day and the MOLISA metric (13,14). The MOLISA (Ministry of Labour, Invalids and Social Affairs) metric is used for determining eligibility for the national anti-poverty program and uses income as an indicator.

Statistical Analysis

Asset indices were calculated using principal component analysis (PCA) of six variables: the presence of clay floors, wood as fuel for cooking, ownership of a stereo system, television, motorbike, or car. From the 2017 survey, the presence of a fridge, computer, air conditioner, washing machine and water heater was also included in the survey. We restricted the asset indices to the same six consumption categories in 2007 and 2017 (24). Using the index, households were divided into groups of their relative wealth and differences in disease prevalence compared between these groups. We assigned consumption data responses as provided by the self-declared head of the household to all members of the household. To adjust for the relative sampling probability of each participant, we used the survey sampling weights based on age, gender, cluster size, areas and post-stratification. Data were analysed using STATA 16.1 and RStudio 1.3.1093.

The distribution of disease between SEP groups is represented by constructing illness concentration curves (25). These are used to quantify whether inequality in SEP exists for a health sector variable, such as TB prevalence (26). We then quantified the position of the geometric mean on the curve by estimating the concentration index, which is defined as twice the area between the concentration curve and the line of equality (the 45-degree line on the graph) (27).

The relationships between TB prevalence, subclinical TB prevalence and SEP are not only explained by individual level risks, but also by interactions between hierarchical levels including the household and wider neighbourhood. In our analyses, we investigated the association between the change in TB prevalence, relative household SEP and absolute provincial poverty (28). We used log-binomial models to examine dependencies between variables nested in each group. We used multilevel models (MLM) with group- and individual-level intercepts as random effects. MLMs aim to explain the association between tuberculosis prevalence over time while taking into account that poverty and the risk of contracting tuberculosis is clustered geographically and in households. MLMs allow us to analyse how the neighbourhood effects explain variation in change in TB prevalence over time.

By partially pooling varying coefficients, we quantified the relationship between variables where we expected the coefficients to vary between neighbourhoods. The Hausman test was used to test the correlation between random error and individual effects (regressors) in the model (see S1 Text).

Ethics

The Vietnam national TB prevalence surveys were approved by the National Hospital for Tuberculosis and Respiratory Diseases in Hanoi (2007) and the Institutional Review Board of the Vietnam National Lung Hospital in 2017 (62/17/CTHKH). This analysis was approved by the ethics committee of the London School of Hygiene and Tropical Medicine (16396).

Results

The characteristics of study participants are summarised in Table 1. Data from 155 919 participants were included in the study, 94 156 from survey one and 61 763 from survey two, of which, 0.23% (218/ 94 156) in survey one and 0.20% (124/ 61 763) in survey two had microbiologically confirmed tuberculosis. Of the patients with confirmed tuberculosis, 21.6% (47/ 218) and 29.0% (36/ 124) reported no cough therefore considered to have subclinical tuberculosis. The average age of study participants was 40.1 and 46.6 years old respectively. The gender balance was similar between the two surveys with 54.8% (51 560/ 94 156) of survey one participants and 56.0% (34 613/ 61 763) of survey two participants being male. Similar proportions of patients between the two surveys reported at least one TB-associated symptom: 21.7% (20 474/ 94 156) in survey one and 19.3% (11 917/ 61 763) in survey two, and episodes of previous TB treatment were similar with 1.3% (1 228/ 94 156) in survey one and 1.3% (789/ 61 763) in survey two.

When comparing household socioeconomic position (SEP) between surveys one and two, a greater proportion of households were in the lowest SEP category 35.1% (19 739/ 56 260) in the 2017 compared to the 2007 survey 24.9% (22 677/ 90 975). This measure is not consistent with the comparison of the absolute wealth estimate (AWE) which is similar between the two surveys. The AWE per household is based on the household SEP, country measures of production and the distribution of wealth between rich and poor. Therefore, these measures are related, but the AWE can be compared between time periods. The mean AWE for survey one was US$ 2 403.80 (SD: 27.0) and for survey two was US$ 2 399.60 (SD: 26.0).

The proportion of households sampled from the Central region of Viet Nam were slightly more in survey two compared to survey one (21.9% versus 15.6%), and the types of housing areas in survey two included more urban (30.2% versus 28.0%) and rural areas (44.1% versus 42.8%) than remote (25.7% versus 29.2%). The percentage of households below the poverty line (living on less than US $2 per day) was 22.0% (SD: 14.6) in 2007 compared to 21.6% (SD: 15.9) in 2017.

Table 1. Comparison of the characteristics of study participants between survey one and survey two.

	Survey one (2007)		Survey two (2017)	
	%	Participants n/N	%	Participants n/N
Tuberculosis (microbiologically confirmed)	0.23%	218/ 94 156	0.20%	124/ 61 763
Percentage of patients with confirmed tuberculosis, sub-clinical	21.6%	47/ 218	29.0%	36/ 124
Individual				
Age				
15 – 24	22.2%	20 934/ 94 156	10.6%	6 542/ 61 763
25 – 34	19.8%	18 681/ 94 156	16.5%	10 191/ 61 763
35 – 44	21.0%	19 790/ 94 156	18.6%	11 508/ 61 763
45 – 54	17.3%	16 285/ 94 156	21.5%	13 289/ 61 763
55 – 64	8.6%	8 138/ 94 156	18.0%	11 143/ 61 763
> 65	11.0%	10 328/ 94 156	14.7%	9 090/ 61 763
Sex				
Male	54.8%	51 560/ 94 156	56.0%	34 613/ 61 763
Female	45.2%	42 596/ 94 156	44.0%	27 150/ 61 763
Of all participants, proportion with at least one TB-associated symptom	21.7%	20 474/ 94 156	19.3%	11 917/ 61 763
Previous TB treatment	1.3%	1 228/ 94 156	1.3%	789/ 61 763
Household				
Absolute wealth estimate, mean (SD)		US$ 2 403.8 (27)		US$ 2 399.6 (26)
Household socioeconomic position				
Lowest	24.9%	22 677/ 90 975	35.1%	19 739/ 56 260
Lower Middle	34.5%	31 419/ 90 975	25.3%	14 207/ 56 260
Upper Middle	16.8%	15 284/ 90 975	22.7%	12 777/ 56 260
Highest	23.7%	21 595/ 90 975	17.0%	9 537/ 56 260
Region				
North	48.5%	45 669/ 94 156	41.4%	25 575/ 61 763
Centre	15.6%	14 646/ 94 156	21.9%	13 525/ 61 763
South	35.9%	33 841/ 94 156	36.7%	22 663/ 61 763
Type of residence				
Urban	28.0%	26 353/ 94 156	30.2%	18 656/ 61 763
Remote	29.2%	27 532/ 94 156	25.7%	15 882/ 61 763
Rural	42.8%	40 271/ 94 156	44.1%	27 225/ 61 763
Province				
Provincial poverty headcount % (2009), mean (SD)		22.0 (14.6)		21.6 (15.9)

Where SD is the standard deviation, a measure of dispersion of the mean.

[INSERT FIGURE 2]

[INSERT FIGURE 3]

Figure 2 shows the proportion of study participants with microbiologically confirmed tuberculosis by SEP for each of the surveys (2007 and 2017). A shift in the distribution of TB disease from a left leaning slope where TB is concentrated among the poor to a right-leaning slope (concentrated among the wealthy) is observed. The proportion of participants from each of the surveys who are represented by each of the SEP groups is shown in Figure 3. In 2007, there is a similar proportion of households in each of the SEP groups. Conversely, in 2017, a greater proportion of households were categorised based on their consumption data as relatively poor rather than wealthy.

[INSERT FIGURE 4]

[INSERT FIGURE 5]

In Figure 4, we show illness concentration curves which represent the cumulative TB prevalence ordered by SEP, relative to the equal distribution line (red dash on the graph). The concentration curve for TB prevalence lies above the equal distribution line, therefore TB prevalence is concentrated among poorer households in the 2007 survey. In the 2017 survey, the concentration curve lies below the equal distribution line indicating that TB prevalence has become more equitably distributed among the population with a higher concentration of TB in wealthier patients. These results are supported by the estimates of concentration indices (see Figure 5). In the 2007 survey, the illness (TB) concentration index was -0.10 (95%CI: -0.08, -0.16, p-value=0.003) and 0.066 (95%CI: 0.06, 0.18, p-value=0.158) in 2017. When we restrict the case definition to subclinical TB, we see similar results though a more pronounced shift towards the wealthier groups in 2017.

Table 2. Associations between individual and household-level variables and tuberculosis (TB) prevalence at each timepoint (2007 and 2017).

	2007 survey		2017 survey	
TB prevalence	PR (95% CI)	p-value	PR (95% CI)	p-value
Age				
15-24	Ref		Ref	
25-34	1.04 (0.26; 1.81)	0.009	1.19 (-0.06; 2.43)	0.063
35-44	1.83 (1.12; 2.54)	<0.001	1.90 (0.71; 3.08)	0.002
45-54	2.05 (1.35; 2.76)	<0.001	2.10 (0.93; 3.27)	<0.001
55-64	2.36 (1.63; 3.09)	<0.001	2.57 (-0.51; 0.62)	<0.001
>=65	2.79 (2.09; 3.49)	<0.001	2.81 (1.64; 3.97)	<0.001
Gender				
Female	Ref		Ref	
Male	1.61 (1.29; 1.92)	<0.001	1.59 (1.25; 1.92)	<0.001
Region				
North	Ref		Ref	
Centre	-0.34 (-0.77; 0.98)	0.129	0.34 (-0.42; 0.72)	0.081
South	0.19 (-0.08; 0.47)	0.170	0.64 (0.30; 0.98)	<0.001
Type of residence				
Urban	Ref		Ref	
Rural	0.08 (-0.23; 0.39)	0.600	-0.46 (-0.79; -0.14)	0.005
Remote	-0.16 (-0.53; 0.20)	0.387	-0.09 (-0.46; 0.29)	0.644
Household socioeconomic position				
Lowest	Ref		Ref	
Lower Middle	-0.22 (-0.54; 0.10)	0.183	0.08 (-0.30; 0.47)	0.671
Upper Middle	0.19 (-0.17; 0.55)	0.309	0.39 (0.01; 0.76)	0.042
Highest	-0.41 (-0.81; -0.00)	0.048	0.76 (0.36; 1.16)	<0.001
AIC	0.034		0.044	

Prevalence Ratios (PRs) and Confidence Intervals (CIs) are estimated using log-binomial mixed effects statistical models. Coefficients are weighted for stratification (differential cluster size, participation by age and sex, stratification by areas and post stratification weight). AIC refers to the Akaike Information Criterion a measure of model fit with a lower value indicating a better-fit model.

In Table 2, the results of evaluations of the associations between tuberculosis prevalence and individual and household risks for each survey is shown separately. In the 2007 survey we found that older age (PR=2.79, 95%CI: 2.09; 3.49), p-value<0.001) and male gender (PR=1.61, 95%CI: 1.29; 1.92, p-value<0.001) is associated with increased TB prevalence. Remote neighbourhoods are negatively associated with TB prevalence (PR=-0.16, 95%CI: -0.53; 0.20, p-value=0.387) in 2007 and (PR=-0.09, 95%CI: -0.46; 0.29, p-value=0.644) in 2017. The wealthiest households were less likely (PR=-0.41, 95%CI: -0.81; -0.00, p-value=0.048) to have tuberculosis than the poorest households in 2007. These associations are similar in direction in the 2017 survey, except for the associations with household SEP where the wealthiest participants are more likely to have tuberculosis (PR=0.76, 95%CI: 0.76; 1.16, p-value < 0.001).

Table 3. Multilevel analyses examining associations between individual, household and neighbourhood level explanatory variables and change in tuberculosis prevalence

	Model A		Model B		Model C	
	PR (95% CI)	p-value	PR (95% CI)	p-value	PR (95% CI)	p-value
Tuberculosis prevalence						
Time (comparator: 2007)	-0.35 (-0.58; -0.12)	0.003	-0.35 (-0.69; -0.01)	0.041	-0.37 (-0.70; -0.04)	0.030
Individual						
Age						
15-24	Ref		Ref		Ref	
25-34	1.34 (0.60; 2.08)	<0.001	1.75 (0.97; 2.54)	<0.001	1.75 (0.97; 2.53)	<0.001
35-44	1.94 (1.25; 2.64)	<0.001	2.26 (1.46; 3.07)	<0.001	2.26 (1.46; 3.07)	<0.001
45-54	2.12 (1.42; 2.81)	<0.001	2.48 (1.59; 3.38)	<0.001	2.48 (1.58; 3.37)	<0.001
55-64	2.41 (1.70; 3.12)	<0.001	2.77 (1.93; 3.61)	<0.001	2.77 (1.93; 3.61)	<0.001
>= 65	2.73 (2.04; 3.42)	<0.001	3.01 (2.24; 3.77)	<0.001	3.01 (2.24; 3.77)	<0.001
Gender						
male	1.42 (1.17; 1.68)	<0.001	1.33 (1.06; 1.60)	<0.001	1.33 (1.06; 1.60)	<0.001
Region						
North			Ref		Ref	
Centre			-0.02 (-0.65; 0.60)	0.944	-0.02 (-0.65; 0.61)	0.950
South			0.24 (-0.20; 0.67)	0.289	0.23 (-0.21; 0.67)	0.304
Type of residence						
Urban	Ref		Ref		Ref	
Rural	-0.13 (-0.38; 0.12)	0.313	-0.16 (-0.56; 0.24)	0.156	-0.17 (-0.56; 0.23)	0.410
Remote	-0.28 (-0.63; -0.06)	0.107	-0.28 (-0.65; 0.11)	0.429	-0.29 (-0.67; 0.09)	0.138
Household						
Household socioeconomic status (SEP)						
Lowest					Ref	
Lower Middle					0.02 (-0.47; 0.51)	0.931
Upper Middle					0.25 (-0.05; 0.56)	0.104
Highest					0.23 (-0.19; 0.66)	0.287
Household absolute wealth estimate (AWE)			0.004 (-0.00; 0.01)	0.140		
Province						
Provincial poverty rate (2009)	-0.01 (-0.02; 0.01)		0.11 (0.02; 0.70)		0.10 (0.02; 0.68)	

Prevalence ratios (PR) were estimated using multi-level mixed effects models with random intercepts. Model A shows individual-level regressors only, Model B shows individual-level and household level variables while using the absolute wealth estimate to understand the impact of household wealth, while Model C uses a relative measure of household SEP. The provincial poverty rate used is the percentage of the population living below US$2 per day.

The results of the multilevel models are shown in Table 3. We present the results of three mixed effects models with random intercepts. Model A is used to investigate the association between individual characteristics and TB prevalence while controlling for time and the provincial poverty rate which is the measured as the percentage of the population below the poverty line. In Model B, we control for the district poverty rate as well as the absolute wealth of the household. While in Model C, the district and relative SEP of households are controlled for to understand how individual and household risks explain the change in TB prevalence between the two surveys. We find that the difference in TB prevalence over time (effect size) reduces when we include indicators of household SEP and provincial poverty which suggests that some of the observed change in TB prevalence can be explained by changes in provincial poverty.

Discussion

We found that in the context of rapid economic growth and equitable distribution of resources in Viet Nam, there was a shift in the distribution of TB from being concentrated among the poor to a more equal distribution among households of different SEP. In the 2007 survey, older age, being male and living in an urban centre was associated with TB prevalence. In the 2017 survey, the association between older age and TB prevalence reduced with urban living. Multi-level models showed the importance of neighbourhood poverty in explaining some of the change in TB prevalence observed.

Studies investigating the association between reductions in TB incidence and economic development have been conducted in a range of settings (1–3,5,7,29). Relationships between economic development and TB prevalence are challenging to examine given distal relationships that influence the causal pathway. Economic development may be measured as an increase in country GDP which represents market productivity, but this is only one aspect of economic development. If economic development increases wealth inequality in a population, patients' vulnerability to TB disease may increase (7). The role of improved healthcare in mediating that relationship is unclear. In a multi-country analysis, Dye et al showed that rates of decline in TB incidence was associated with biological, social and economic determinants (1). Focusing on poverty alleviation and social protection policies, Carter et al. 2018 found that reducing extreme poverty may reduce the global incidence of tuberculosis by 33%, simultaneously expanding social protection coverage may reduce incidence by 84% (2). While Dye et al. found that health service programmatic indicators did not explain the reduction in TB incidence, Reeves et al. found that reductions in public spending (through economic recession) reduced spending on tuberculosis control and argued that this may lead to increased TB prevalence (3). These studies examined associations between different components of economic development and tuberculosis prevalence, but empirical data were limited. Conversely, Siroka et al. 2016 used TB prevalence survey data from eight countries to examine the association between household level poverty and TB prevalence (6). The study was cross-sectional and did not find a consistent association between household SEP and TB prevalence. From these studies we therefore understand that it is possible that the relationship between economic development and change in tuberculosis prevalence is not simply dependent on the household or on investment in the health services but rather on a combination of risk factors across neighbourhood interactions.

However, economic growth and reduction in poverty may not be the only explanation for the change as there were also improvements in TB diagnostics and health service access through an expansion of health insurance in Viet Nam. Possible explanations for the results of our study therefore include that the rapid economic development in Viet Nam led to TB patients being wealthier in the second survey.

However, this is mediated by lower participation in the second survey of wealthy households because of the expansion of the Vietnamese National Health Insurance, making free health check-ups for participation in a TB prevalence survey less attractive (1,3). Despite lower participation from relatively wealthier households, we found that TB burden was more concentrated among the wealthy in the 2017 survey than in 2007 suggesting selective participation.

We found that the relative household SEP was weakly associated with TB prevalence after controlling for known individual-level risk factors such as age and gender. (30) Our finding related to the trend towards tuberculosis being less concentrated among the poor when measured over time corresponds to the findings of Ataguba et al in South Africa (24). However, in South Africa, economic development has been accompanied by persistently high levels of income inequality and the effect was likely mediated by the expansion of the ART programme which disproportionally will have benefitted the poor. Our findings suggest that neighbourhood-level (provincial) poverty explains much of the variation in TB prevalence over time. Neighbourhood level poverty may be a signal of fewer economic opportunities and therefore a greater vulnerability to tuberculosis (7). Our measure of household level SEP was primarily based on consumption data collected during the prevalence survey and some of the important factors predicting poverty in Viet Nam such as education were not included in this measure. Furthermore, consumption data are sensitive to change over time, for example an item that was a signal of prosperity in 2007 may no longer be a good indicator of wealth in 2017. However, we used different household and neighbourhood level measures of poverty including rural residences, the region where the district is situated with Viet Nam as well as the percentage of people in the district who are considered poor (the district poverty rate). We also estimated the absolute wealth of households and the primary results of the study held across the different measurements used. While TB prevalence surveys are valuable for understanding socio-economic differences in TB prevalence in countries, given that TB is a relatively rare disease in the population studied, there is limited power to explore the socio-economic drivers of tuberculosis prevalence (6). However, combining repeated cross-sectional surveys with neighbourhood deprivation estimates during a period of remarkable economic growth provides valuable insights into the dynamics of the relationships between TB and economic development in Viet Nam.

Conclusions

This is the first study to use repeat direct measurements of TB burden to empirically examine the relationship between equitable economic development and a reduction in TB prevalence. We found that with equitable economic growth and a reduction in TB burden, TB became less concentrated among the poor in Viet Nam.

References

1. Dye C, Lönnroth K, Jaramillo E, Williams B, Raviglione M. Trends in tuberculosis incidence and their determinants in 134 countries. Bull World Health Organ. 2009 Sep 1;87(9):683–91.

2. Carter DJ, Glaziou P, Lönnroth K, Siroka A, Floyd K, Weil D, et al. The impact of social protection and poverty elimination on global tuberculosis incidence: a statistical modelling analysis of Sustainable Development Goal 1. Lancet Glob Health. 2018 May;6(5):e514–22.

3. Reeves A, Basu S, McKee M, Sandgren A, Stuckler D, Semenza JC. Tuberculosis control and economic recession: longitudinal study of data from 21 European countries, 1991–2012. Bull World Health Organ. 2015 Jun 1;93(6):369–79.

4. Reeves A, Basu S, McKee M, Stuckler D, Sandgren A, Semenza J. Social protection and tuberculosis control in 21 European countries, 1995–2012: a cross-national statistical modelling analysis. Lancet Infect Dis. 2014 Nov;14(11):1105–12.

5. Siroka A, Ponce NA, Lönnroth K. Association between spending on social protection and tuberculosis burden: a global analysis. Lancet Infect Dis. 2016 Apr;16(4):473–9.

6. Siroka A, Law I, Macinko J, Floyd K, Banda RP, Hoa NB, et al. The effect of household poverty on tuberculosis. 2018;12.

7. Lönnroth K, Jaramillo E, Williams BG, Dye C, Raviglione M. Drivers of tuberculosis epidemics: The role of risk factors and social determinants. Soc Sci Med. 2009 Jun;68(12):2240–6.

8. Andrews J. The epidemiological advantage of preferential targeting of tuberculosis control at the poor. Int J Tuberc Lung Dis. 2014;19(4):375–80.

9. Hoa NB, Sy DN, Nhung NV, Tiemersma EW, Borgdorff MW, Cobelens FG. National survey of tuberculosis prevalence in Viet Nam. Bull World Health Organ. 2010 Apr 1;88(4):273–80.

10. Nguyen HV, Tiemersma EW, Nguyen HB, Cobelens FGJ, Finlay A, Glaziou P, et al. The second national tuberculosis prevalence survey in Vietnam. Schwartzman K, editor. PLOS ONE. 2020 Apr 23;15(4):e0232142.

11. Nguyen HV, Nguyen HB, Nguyen NV, Cobelens F, Finlay A, Dao CH, et al. Decline of Tuberculosis Burden in Vietnam Measured by Consecutive National Surveys, 2007–2017. Emerg Infect Dis. 2021 Mar;27(3):872–9.

12. Lee H-Y, Oh J, Hoang VM, Moon JR, Subramanian SV. Use of high-level health facilities and catastrophic expenditure in Vietnam: can health insurance moderate this relationship? BMC Health Serv Res. 2019 Dec;19(1):318.

13. The World Bank Data: Viet Nam [Internet]. [cited 2020 Dec 21]. Available from: https://data.worldbank.org/country/VN

14. The World Bank. The World Bank mapVIETNAM dataset [Internet]. Available from: http://www5.worldbank.org/mapvietnam/

15. Krieger N, Williams D, Moss N. Measuring social class in US public health research: concepts, methodologies and guidelines. Annu Rev Public Health [Internet]. [cited 2021 Apr 19]; Available from: https://pubmed.ncbi.nlm.nih.gov/9143723/

16. Gelman A, Hill J, Vehtari A. Regression and other stories. Cambridge, UK: Cambridge University Press; 2021.

17. Kirkwood BR, Sterne JAC, Kirkwood BR. Essential medical statistics. 2nd ed. Malden, Mass: Blackwell Science; 2003. 501 p.

18. Pedrazzoli D. Modelling the social and structural determinants of tuberculosis: opportunities and challenges. Int J Tuberc Lung Dis. 2017;21(9):957–64.

19. Rothman KJ, Greenland S, Lash TL. Modern Epidemiology. Third. Lippincott Williams & Wilkins; 2008.

20. Krieger N. Theories for social epidemiology in the 21st century: an ecosocial perspective. Int J Epidemiol. 2001 Aug;30(4):668–77.

21. Esmail H, Dodd PJ, Houben RMGJ. Tuberculosis transmission during the subclinical period: could unrelated cough play a part? Lancet Respir Med. 2018 Apr;6(4):244–6.

22. Frascella B, Richards AS, Sossen B, Emery JC, Odone A, Law I, et al. Subclinical tuberculosis disease - a review and analysis of prevalence surveys to inform definition, burden, associations and screening methodology. Clin Infect Dis. ciaa1402.

23. Nguyen, Hai. The decline of tuberculosis burden in Vietnam: a comparison of two consecutive national surveys. TBC.

24. Ataguba JE, Akazili J, McIntyre D. Socioeconomic-related health inequality in South Africa: evidence from General Household Surveys. Int J Equity Health. 2011;10(1):48.

25. Jann B. Estimating Lorenz and concentration curves. Stata J. 2016;16(4):837–66.

26. World Bank, editor. Chapter 8: Concentration Index. In: Analyzing health equity using household survey data [Internet]. Available from: https://www.worldbank.org/en/topic/health/publication/analyzing-health-equity-using-household-survey-data

27. Erreygers G, Van Ourti T. Measuring socioeconomic inequality in health, health care and health financing by means of rank-dependent indices: A recipe for good practice. J Health Econ. 2011 Jul;30(4):685–94.

28. Atkinson AB. Measuring poverty around the world. Princeton, NJ: Princeton University Press; 2019. 429 p.

29. Hermans S, Horsburgh Jr. CR, Wood R. A Century of Tuberculosis Epidemiology in the Northern and Southern Hemisphere: The Differential Impact of Control Interventions. Dheda K, editor. PLOS ONE. 2015 Aug 19;10(8):e0135179.

30. Horton KC, Sumner T, Houben RMGJ, Corbett EL, White RG. A Bayesian Approach to Understanding Sex Differences in Tuberculosis Disease Burden. Am J Epidemiol. 2018 Nov 1;187(11):2431–8.

Click here to access/download;Figure;figures.pdf

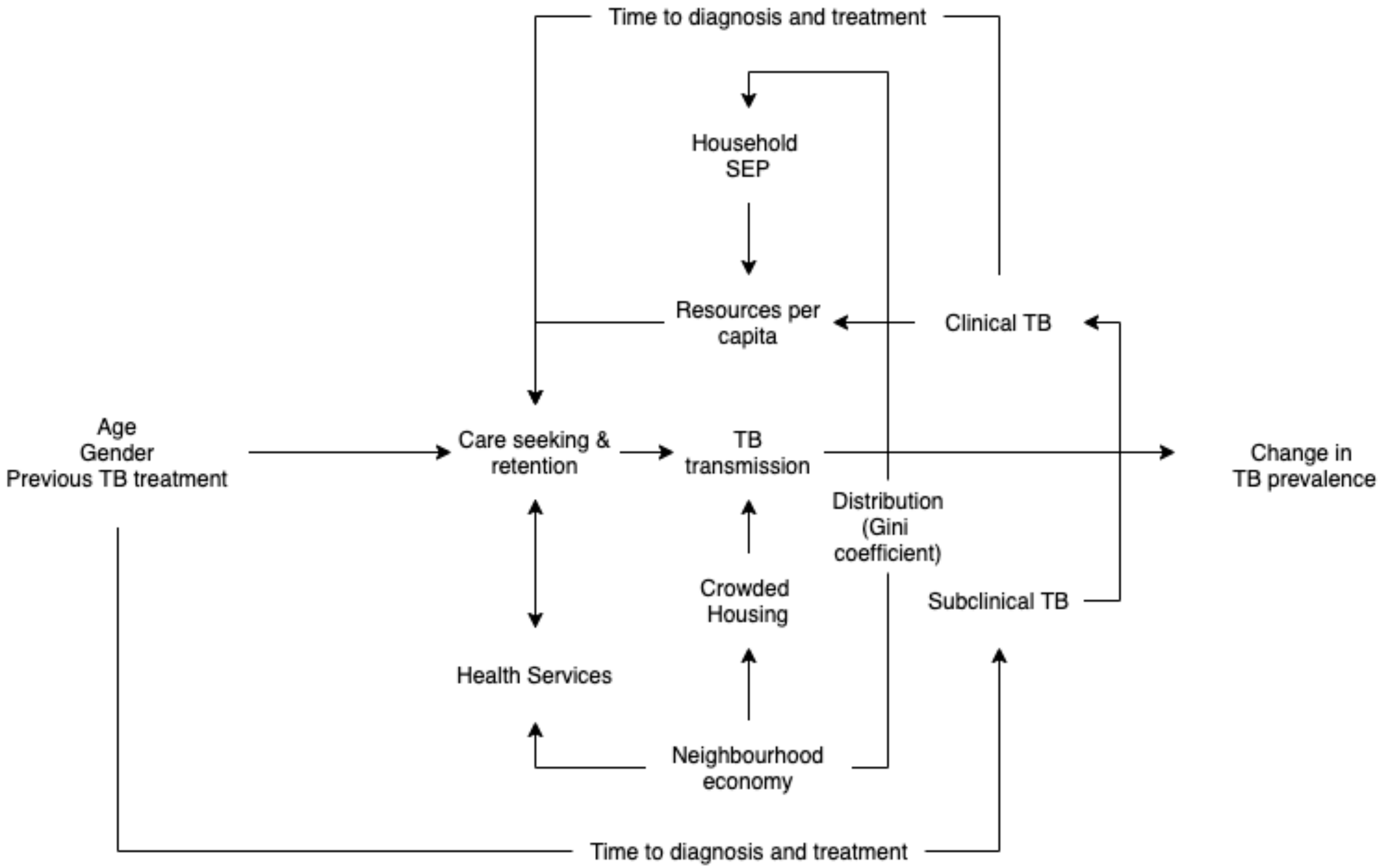

Figure 1. Causal diagram of social determinants of TB prevalence in Viet Nam.

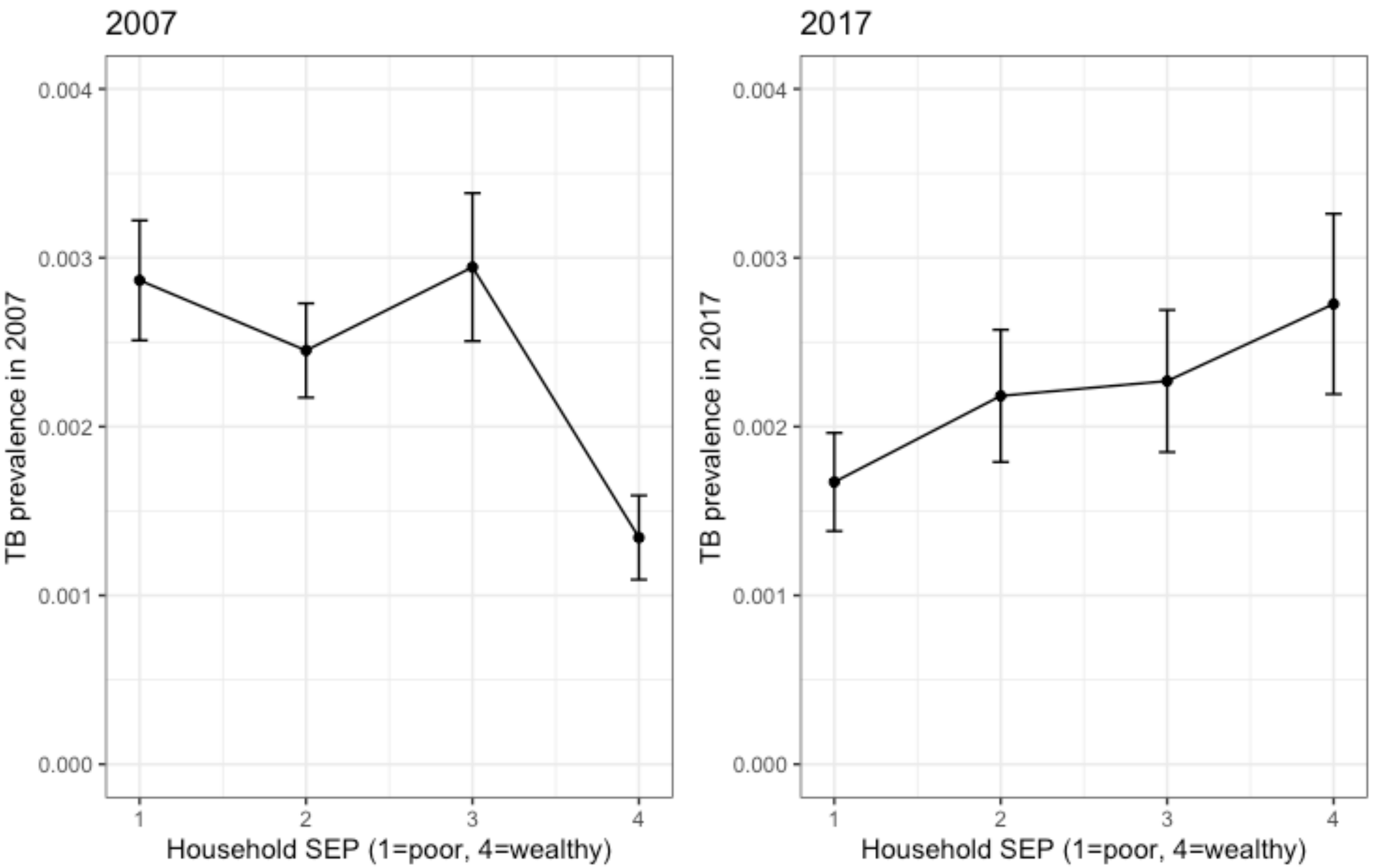

Figure 2. The distribution of tuberculosis prevalence by socioeconomic position (SEP) as measured in the 2007 and 2017 tuberculosis prevalence surveys.

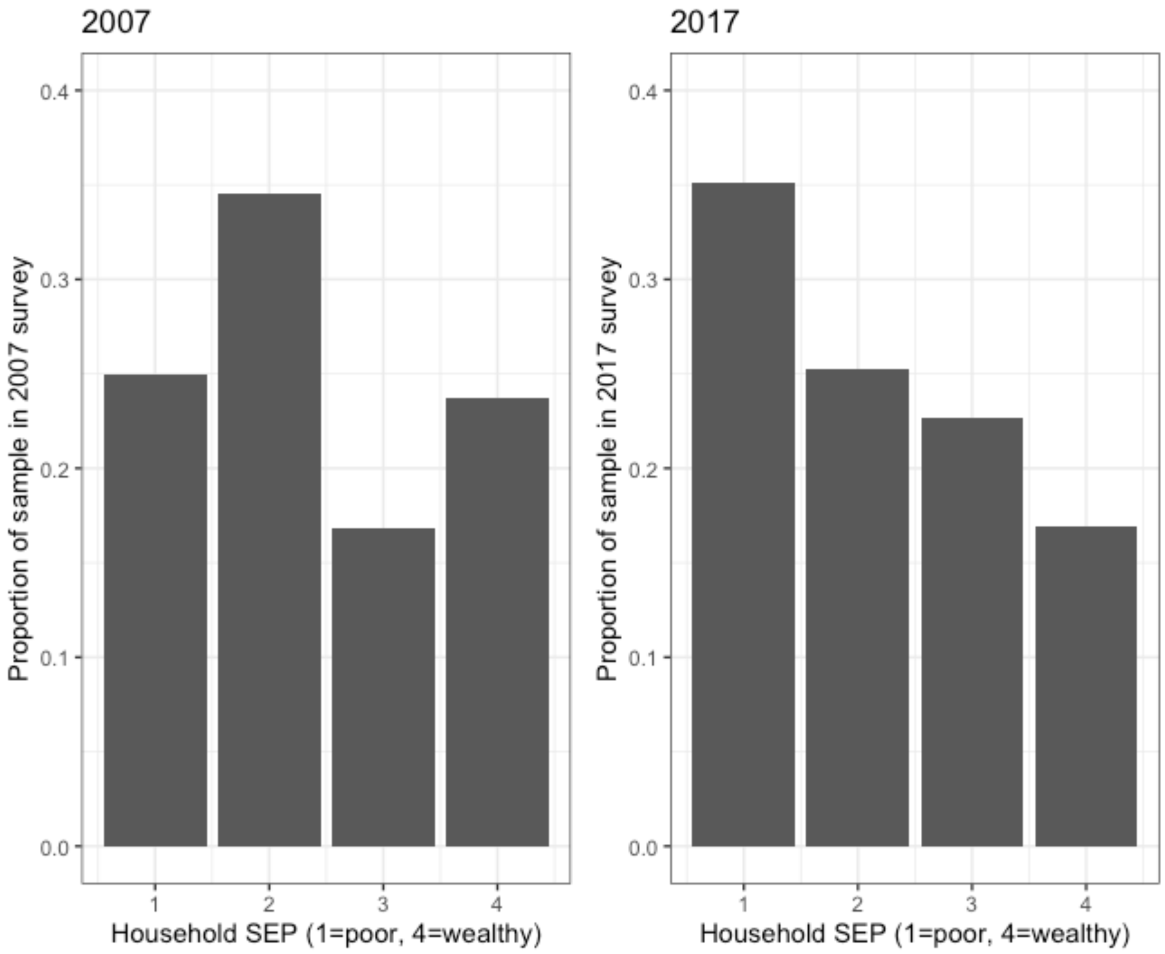

Figure 3. Proportion of participants by socioeconomic position (SEP) in 2007 and 2017.

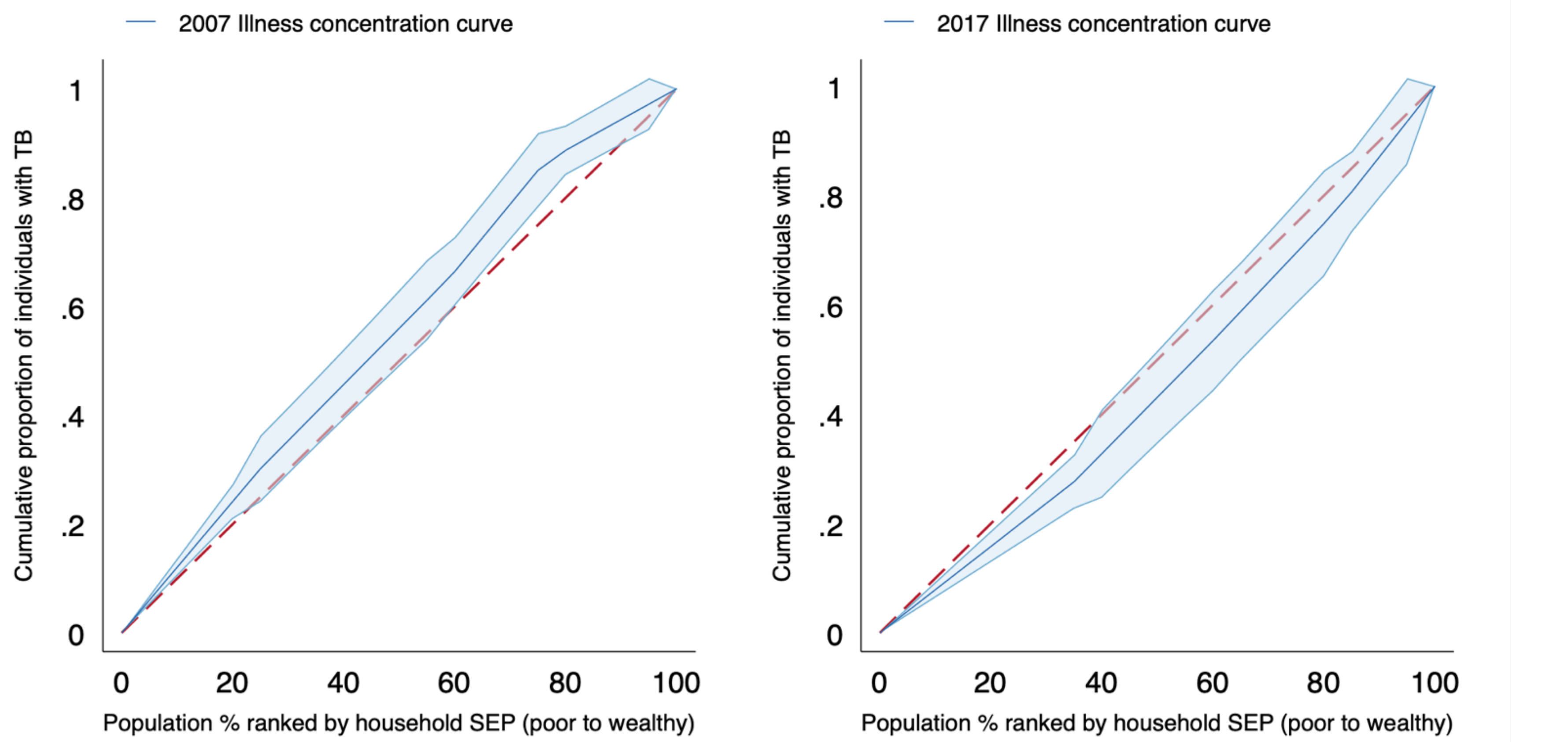

Figure 4. Illness concentration curves. The straight line (red dash) represents the equal distribution line, while the blue curve is the cumulative TB prevalence in the population ranked by assets. The blue shaded area is the uncertainty interval. A curve above the equal distribution line means that TB is concentrated among the poor and a curve below the equal distribution line means that TB is concentrated among the wealthy. Concentration curves for TB associated symptoms are included in S1 Text.

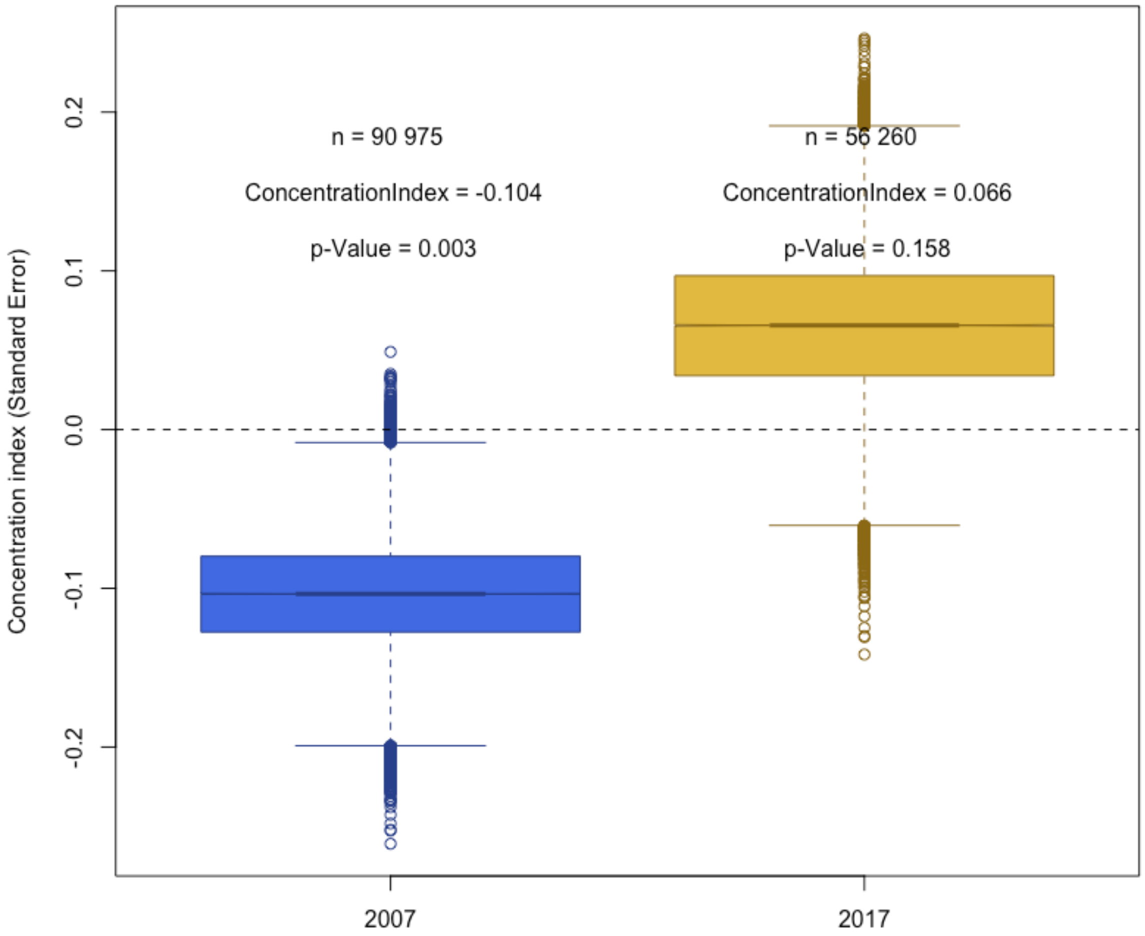

Figure 5. Illness concentration indices (ConcIn) for 2007 and 2017 Viet Nam TB prevalence surveys. Sampling weights were applied. A negative concentration index means that the health outcome (tuberculosis illness) is concentrated in the poor, while a positive index value means that the disease in concentrated in those who are wealthier. The concentration index (Figure 4) is an expression of the area between the concentration curve (Figure 3) and the line of perfect equality.

S1 Text

Click here to access/download
Supporting Information
s1 text.pdf
