# Supplementary material for: Social determinants of the changing tuberculosis prevalence in Việt Nam: Analysis of population-level cross-sectional studies
Source: PLoS Med. 2022 Mar 18;19(3):e1003935. doi: 10.1371/journal.pmed.1003935 (PMC8932606; doi:10.1371/journal.pmed.1003935)
Supplement: Attachment — Submitted filename: responses V2.docx [file pmed.1003935.s004.docx]

Dr Nicola Foster

Department of Infectious Disease and Epidemiology

London School of Hygiene and Tropical Medicine

Keppel street, London WC1E 7HT

[nicola.foster@lshtm.ac.uk](mailto:nicola.foster@lshtm.ac.uk)

25 November 2021

Dear Dr Raffaella Bosurgi,

**RE: Manuscript PMEDICINE-D-21-03248R1 "Social determinants of the changing tuberculosis prevalence in Việt Nam: analysis of population-level cross-sectional studies"**

Our responses to each of the four reviewers as well as the editorial team are collated below, grouped by manuscript section and/or topic.

In revising the manuscript for further consideration, your revisions should address the specific points made by each reviewer and the editors. Please also check the guidelines for revised papers at [http://journals.plos.org/plosmedicine/s/revising-your-manuscript](http://protect-eu.mimecast.com/s/xkEGC5LPGCWqgJouz0GHI?domain=journals.plos.org) for any that apply to your paper. In your rebuttal letter you should indicate your response to the reviewers' and editors' comments, the changes you have made in the manuscript, and include either an excerpt of the revised text or the location (eg. page and line number) where each change can be found. Please submit a clean version of the paper as the main article file; a version with changes marked should be uploaded as a marked-up manuscript. In addition, we request that you upload any figures associated with your paper as individual TIF or EPS files with 300dpi resolution at resubmission; please read our figure guidelines for more information on our requirements: [http://journals.plos.org/plosmedicine/s/figures](http://protect-eu.mimecast.com/s/2aMEC69QJSGQVNEs6ihCJ?domain=journals.plos.org). While revising your submission, please upload your figure files to the PACE digital diagnostic tool, [https://pacev2.apexcovantage.com/](https://protect-eu.mimecast.com/s/4HoCC7XRKiW3EgvuBlrci?domain=pacev2.apexcovantage.com). PACE helps ensure that figures meet PLOS requirements. To use PACE, you must first register as a user. Then, login and navigate to the UPLOAD tab, where you will find detailed instructions on how to use the tool. If you encounter any issues or have any questions when using PACE, please email us at [PLOSMedicine@plos.org](mailto:PLOSMedicine@plos.org).

# **General**

## Style revisions

1. Please revise your title according to PLOS Medicine's style.
2. I strongly suggest to the authors to follow the PLOS Medicine's submission guidelines. Please use continuous line numbers, do not restart the numbering on each page. In the text, cite the reference number in square brackets according to the PLOS Medicine's submission guidelines.
   Please, organize the level 1 and 2 heading in the manuscript body according to [https://journals.plos.org/plosmedicine/s/file?id=9cba/PLOS%20Manuscript%20Body%20Formatting%20Guidelines.pdf](https://protect-eu.mimecast.com/s/LlXMCmy54uRE1xZswGEF1?domain=journals.plos.org)

The manuscript has been reformatted to comply with the PLOS manuscript body formatting guidelines.

## **Editorial**

3. The Data Availability Statement (DAS) requires revision. Please include an appropriate weblink to the LSHTM Data Compass website.

The data is available on LSHTM Data Compass (<https://doi.org/10.17037/DATA.00002373>), and the data sharing statement has been updated to the following:

“Data analysed in this study was provided by the Việt Nam National Lung Hospital, subject to the signing of an agreement that the data is kept confidential and is not made available to others. Researchers wishing to use the data should apply to Dr Hoa and the Institutional Research Board at the Việt Nam National Lung hospital by emailing [bvptw@bvptw.org](mailto:bvptw@bvptw.org). A description of the dataset, and an overview of variables analysed plus the code required to produce the analysis may be found at <https://doi.org/10.17037/DATA.00002373>.”

4. I suggest reading the manuscript carefully, since there are many little mistakes that could be solved easily by the authors. The manuscript requires professional editing in the English language to make it easy to read and understand.

The manuscript has been proofread and errors corrected.

5. I strongly suggest to the authors the Strengthening the Reporting of Observational Studies in Epidemiology (STROBE) Statement: Guidelines for Reporting Observational Studies.
   It is available from von Elm E, Altman DG, Egger M, Pocock SJ, Gøtzsche PC, Vandenbroucke JP, et al. (2007) The Strengthening the Reporting of Observational Studies in Epidemiology (STROBE) Statement: Guidelines for Reporting Observational Studies. PLoS Med 4(10): e296. [https://doi.org/10.1371/journal.pmed.0040296](https://protect-eu.mimecast.com/s/IzDxCnO14c6MlEzczxPnw?domain=doi.org)

We have revised the reporting of the study to be in line with the recommendations of the STROBE checklist. The completed checklist is included in this submission as a supplementary appendix (S2 – STROBE checklist).

6. I also suggest to the authors to verify the United to End TB: Every Word Counts launched by Stop TB Partnership. Available from: [http://www.stoptb.org/assets/documents/resources/publications/acsm/LanguageGuide_FtorWeb20131110.pdf](http://protect-eu.mimecast.com/s/TSdOCoy14uKxlnMcmzkfI?domain=stoptb.org)
   The language guide supports the call for change in the upcoming Global Plan to End TB 2016-2020, which includes changing the mindset, language, and dialogue on TB as one of the key paradigm shifts required to reach the End TB goals.

The language used in the manuscript has been amended based on the language guide. Thank you for drawing our attention to this.

7. To help us extend the reach of your research, please provide any Twitter handle(s) that would be appropriate to tag, including your own, your co-authors’’, your institution, funder, or lab.

The appropriate Twitter handles have been added to the title page:

“Twitter: @DrNicolaFoster; @matthew_quaife; @Rein_Houben; @ViệtNhung1; @lshtm_tbmod; @LSHTM_TB”

## **Causal inference**

8. Your study is observational and therefore causality cannot be inferred. Please remove language that implies causality throughout the manuscript, such as “determinants”, “contributed to”, “explanation for”, and so forth. Refer to associations instead.
   Please avoid assertions of primacy ("We report for the first time....") by stating, “to our knowledge” or similar

Text has been amended: “To our knowledge, this is the first study to use repeat direct measurements of tuberculosis burden to empirically examine the relationship between equitable economic development and a reduction in tuberculosis prevalence.”

9. First, there is the general issue of causal language. This is an observational study and causality cannot be inferred. Even in the title the word "determinants" is used. Not only should causal words be changed, but the "causal pathways" section should make it clear that these are *possible* causal pathways.

While there have been improvements in the application of causal inference to observational studies, we agree that a limitation of this study is the lack of a valid comparator to investigate a causal relationship between economic development and changes in tuberculosis prevalence. We have therefore amended the language throughout the study to clarify this limitation. Where we are referring to the social determinants of disease, we are using the phrase to signal a body of work and recognised definitions. We clarified our use of the terminology in the following way:

“Social determinants of health refer to the socio-economic, cultural and political aspects in the community that affects the health of populations [25]. These determinants include people’s living - and working conditions, water and sanitation, housing, unemployment as well as the political drivers of health. An individual’s biological risk of developing TB is influenced by age, gender and previous treatment and how these intersect with household risk and household economic position in the neighbourhood [26].”

# **Title and title page**

10. Your title must be nondeclarative and not a question. It should begin with main concept if possible. Please place the study design in the subtitle (i.e., after a colon). For example, “Changing tuberculosis prevalence by socioeconomic status in Viet Nam: Analysis of population-level cross-sectional studies.”
11. I suggest to the authors the title "Social determinants and changing prevalence in decrease of the burden of tuberculosis in Vietnam".

The study title has been amended.

12. There is unnecessary information in the title page. Please, follow the PLOS Medicine's submission guidelines([https://journals.plos.org/plosmedicine/s/file?id=3fac/PLOS%20Affiliations%20Formatting%20Guidelines.pdf](https://protect-eu.mimecast.com/s/i5bbCp215ixXQ2Gi9Ngqu?domain=journals.plos.org)).
13. line 21 - 23: Delete these information
14. Line 32 - 36: Delete these information
15. Line 1 - 3: Delete these information

The additional information has been deleted and the manuscript has been reformatted based on the PLOS Medicine submission guidelines.

## **Language**

16. Line 2: Please, replace the word "Viet Nam" by "Vietnam", according to the English spelling in the whole manuscript body.

We use the spelling of the country name as used by the government of Viet Nam in official documents. We note that the United Nations similarly use “Viet Nam” as the spelling of the country name. When thinking about this more explicitly as during the response to the review, we wanted to take this approach further and use the spelling of the country name as used by citizens of the country. As a result, we include the phonetic signs in the name wherever used in the manuscript, Việt Nam.

17. Line 5: Key words are not necessary according to the PLOS Medicine's submission guideline.

Key words have been removed.

18. Page 3: Please delete the author summary heading. This section is not necessary in the manuscript and it does not follow the PLOS Medicine's submission guidelines. Merge this information in the background section, methods section, discussion section and conclusion section.

Author summaries are published in PLOS Medicine and is a summary which increases the accessibility of the manuscript, we would therefore prefer to include a summary.

# **Abstract**

19. Please structure your abstract using the PLOS Medicine headings (Background, Methods and Findings, Conclusions).

- Please combine the Methods and Findings sections into one section, “Methods and findings”.
- Please ensure that all numbers presented in the abstract are present and identical to numbers presented in the main manuscript text.
- Please include the important dependent variables that are adjusted for in the analyses.
  In the last sentence of the Abstract Methods and Findings section, please describe the main limitation(s) of the study's methodology.
- Please place the Author Summary after the Abstract.
- The authors must follow the submission guidelines to provide a better abstract of the study. I suggest to the authors to minimize the use of abbreviations.
- Line 10: Please, replace the word ˜Methods" by "Methods and findings".

The abstract has been amended following the PLOS Medicine guidelines and using the relevant headings.

20. Line 27:  Please, replace the word "interpretation" by "conclusion"

Amended.

21. Line 31: Please, delete the "funding" heading.

Amended.

# **Introduction**

22. The Background section should explain the background to the study, its aims, a summary of the existing literature and why this study was necessary or its contribution to the field. There are many blanks about the existing literature about the theme in the manuscript. I strongly suggest an explanation how the Social Determinants of Health impact the prevalence and how they increase the burden of tuberculosis. A deep literature review about the theme is necessary to understand the contribution to the public health field.

We have expanded the literature review and have added a clearer link between the concept of social determinants of health, by adding the following:

“Social determinants of health refer to the socio-economic, cultural and political aspects in the community that affects the health of populations [25]. These determinants include people’s living - and working conditions, water and sanitation, housing, unemployment as well as the political drivers of health. An individual’s biological risk of developing TB is influenced by age, gender and previous treatment and how these intersect with household risk and household economic position in the neighbourhood [26].” (page 5, lines 119-124)

23. Line 1 – 2: Please insert a reference in this statement.

We added the following references in support of this statement: “The relationship between tuberculosis disease (TB) and development is well documented [1–3].”

- Lonnroth K, Jaramillo E, Williams BG, Dye C, Raviglione M. Drivers of tuberculosis epidemics: the role of risk factors and social determinants. Soc Sci Med. 68: 2240–2246. doi:10.1016/j.socscimed.2009.03.041
- de Castro DB, de Seixas Maciel EMG, Sadahiro M, Pinto RC, de Albuquerque BC, Braga JU. Tuberculosis incidence inequalities and its social determinants in Manaus from 2007 to 2016. Int J Equity Health. 2018;17: 187. doi:10.1186/s12939-018-0900-3
- Wingfield T, Tovar MA, Huff D, Boccia D, Saunders MJ, Datta S, et al. Beyond pills and tests: addressing the social determinants of tuberculosis. Clin Med. 2016;16: s79–s91. doi:10.7861/clinmedicine.16-6-s79

24. Line: 2: Replace the word "Introduction" by "Background".

The PLOS MED authorship guidelines requests the use of the term Introduction rather than Background. I have therefore kept the term “Introduction” for the first section of the manuscript.

25. Line 2 - 5: Rewrite the paragraph adding the aim of the study following the scientific writing criteria (verb in the infinite tense).

We have rewritten the paragraph that includes the aim of the study, as well as the preceding paragraph in the following way:

“The increase in GDP per capita with an unchanging Gini coefficient suggests that the benefits of rapid economic development observed in Việt Nam have been distributed equitably among the population, an example of shared prosperity. Given that the individual risk of tuberculosis disease is increased by poor household socio-economic position, the rapid and sustained economic growth in Việt Nam was an opportunity to examine simultaneous changes in tuberculosis prevalence and economic growth.

In the analysis presented here, we use the opportunity of measured longitudinal trends in both poverty and TB burden to examine the individual, household and neighbourhood social determinants of the reduction in TB prevalence in Việt Nam. The aim of this analysis is to estimate the differential prevalence of tuberculosis by Socio-Economic Position (SEP) and to examine the individual, household, and neighbourhood social determinants of the reduction in TB prevalence in Việt Nam.”

26. Line 4: Replace the abbreviation "TB" after the word "tuberculosis".

The word “TB” has been replaced with “tuberculosis” through the manuscript.

27. page 4, ln 4: add a reference

Additional references have been added in support of the statement: “The relationship between tuberculosis disease and development is well documented [1–3].”

28. Line 5: The authors stated: "Ecological studies…", but the authors just cited one reference. Please, insert references in this statement.

The sentence has been expanded with more references added. “Ecological studies analysing historical trends have attributed sustained reductions in the prevalence of tuberculosis to a combination of reductions in crowded living conditions [4], effective anti-tuberculosis chemotherapy [5], and the improvements in housing, access to health services, and nutrition that accompany economic development, poverty reduction and social policy such as social protection [6]. “

29. Line 7 - 10: Please, rewrite this statement and insert a reference.

The World Health Organisation’s End TB strategy and the UN’s Sustainable Development Goals 1 and 3 recognise the importance of healthcare and the control of communicable diseases including tuberculosis, as outcomes of and crucial for economic development [7–9].

30. Page 4, line 23: Give a broader picture of TB in Viet Nam, can do this in one sentence, include national incidence or prevalence

Additional information added, the revised sentence no read as follows: “Việt Nam is an example of a country that has experienced notable sustained economic growth. In 2006, the smear-positive tuberculosis incidence in Việt Nam was estimated to be 260 per 100 000 population, and the treatment success rate was 92% [17].”

31. Line 24: Replace the word "Viet Nam" by "Vietnam".

We use the spelling of the country name as used by the government of Viet Nam in official documents. We note that the United Nations similarly use “Viet Nam” as the spelling of the country name. We further outline our rationale for our approach to the spelling of the country name in point 16.

32. Line 33 - 34: Please insert a reference in this statement.

A reference to the data source has been added.

# **Methods**

33. Did your study have a prospective protocol or analysis plan? Please state this (either way) early in the Methods section: If a prospective analysis plan (from your funding proposal, IRB or other ethics committee submission, study protocol, or other planning document written before analyzing the data) was used in designing the study, please include the relevant prospectively written document with your revised manuscript as a Supporting Information file to be published alongside your study and cite it in the Methods section. A legend for this file should be included at the end of your manuscript. If no such document exists, please make sure that the Methods section transparently describes when analyses were planned, and when/why any data-driven changes to analyses took place. In either case, changes in the analysis - including those made in response to peer review comments-- should be identified as such in the Methods section of the paper, with rationale.

Analyses plans were part of a larger funding proposal, which only described the broad analysis area of socio-economic determinants and subclinical disease. Given that it contains a wider set of unpublished ideas, it is not appropriate to share the full proposal. The methods described in the funding proposal describe the process from the point of engagement with the Việt Nam NTP. From that point onward, no major changes to the approach were made.

34. Please ensure that the study is reported according to the STROBE guideline for observational studies and include the completed STROBE checklist as Supporting Information. Please add the following statement, or similar, to the Methods: "This study is reported as per the Strengthening the Reporting of Observational Studies in Epidemiology (STROBE) guideline (S1 Checklist)." The STROBE guideline can be found here: [http://www.equator-network.org/reporting-guidelines/strobe/](http://protect-eu.mimecast.com/s/DLZ_Ck714TqmYE7I0PcI_?domain=equator-network.org)
35. The authors must follow the STROBE statement to better report the sections in the manuscript body, specially in the methods section.
    Add new headings according to the STROBE statement (study design, setting…)
    The paragraphs do not have a logical sequential that allow to understand the aim of the study.

We made changes to the reporting of the study, following the STROBE guidelines. The reporting checklist is included in the submission as S2 Checklist and the following sentence has been added to the manuscript: “This study is reported as per the Strengthening the Reporting of Observational Studies in Epidemiology (STROBE) guideline (S2 Checklist).”

36. Please clarify whether population case detection rates, treatment success rates, or earlier diagnosis changed during the study period in the country and may have affected prevalence.
    Did you account for population changes in your multilevel models?

Treatment success rates have remained similar, at approximately 93% during the study period. While the focus of the analysis and therefore the manuscript presented here focused on the association between household socio-economic position and the distribution (equity) of tuberculosis burden in the population, a related manuscript based on an analysis of this data discusses the temporal trends in TB prevalence in more details:

Nguyen HV, Nguyen HB, Nguyen NV, Cobelens F, Finlay A, Dao CH, et al. Decline of Tuberculosis Burden in Vietnam Measured by Consecutive National Surveys, 2007–2017. Emerg Infect Dis. 2021;27: 872–879. doi:10.3201/eid2703.204253

37. Page 6, line 4: Add reference 9 and 10 here.

Added.

38. Page 6 ln 17-31: This whole paragraph should be part of the introduction.

This paragraph explains the causal diagram included as Figure 1, which is why it is discussed in this section of the manuscript. However, we have added an expanded overview of the evidence review that were used to develop the causal diagram in the Introduction section of the manuscript.

39. Page 7 ln 4: Add number of households included if you have access to it.

Further details on the sampling strategy and selection of participants were included in Figure S1.1.

40. Page 7, ln 8: Was this done for all participant? e.g Did they all do chest X ray, regardless of symptoms? It not clear

A summary of the sampling and procedures used in both surveys have been added to S1 Text. See Figure S1.1.

41. Page 7, ln 14: Was this done for both 1st and 2nd survey? Or was it only for 2nd survey?

For both surveys, the TB case definition of screen-positive, smear microscopy test and at least one positive LJ culture was used. This was to ensure comparability between the two surveys.

42. Page 7, ln 20: Add more details about data obtained from World bank. level measures of poverty were from which years?

Data from 2009 and 2013 were used, the text was amended to incorporate this in the text: “Data from the prevalence surveys were matched to provincial level measures of poverty using data from the World Bank, the percentage of people living on less than $2 per day in 2009 and the 2013 MOLISA metric [22,23]. The MOLISA (Ministry of Labour, Invalids and Social Affairs) metric is used for determining eligibility for the national anti-poverty program and uses income as an indicator.”

43. line 8-9 Why were these categorized? Categorizing a continuous variable is nearly always a mistake. The index can be used as is. Nonlinearity can be explored with splines. If the authors then want to compare various percentiles, they can do so.

We categorise the asset index variable to compare in the regression analyses how households of similar socio-economic position’s health outcomes compare to other households of higher/ lower socio-economic position.

44. p. 9 line 4 This should really be factor analysis, not PCA. The results are often similar, but the goals are different. The goals here are clearly those of factor analysis - to uncover a latent factor from several measures that are related to it.
    Line 20: Move the meaning of the abbreviation for the first time it appears. 
    In the Statistical Analysis section, why did the authors only use six variables to asset indices?

Factor analysis and Principal Component Analysis (PCA) are similar in that they are both data reduction approaches, and therefore allows you to capture variance in variables in a smaller set for analysis. The difference is that PCA is a linear combination of variables with component identified while factor analysis is used to measure a latent variable (i.e., a variable that cannot be directly measured). PCA is therefore the correct approach to use for the asset index as in this case we are using a variable that is directly measured such as assets. A helpful description of these differences and the analytical implications can be found [here](https://www.theanalysisfactor.com/the-fundamental-difference-between-principal-component-analysis-and-factor-analysis/).

With regards to the decision related to which asset variables to include in the asset index, the decision making related to the development of the asset index is discussed in detail in the supplementary appendix, section 2 of S1 Text. Briefly, the prevalence surveys included slightly different consumption data questions in the 2007 compared to 2017 surveys. Mostly, the 2017 survey included an expanded set of questions. Comparability of these questions between surveys are more complicated than comparing the responses to the same questions, ten years apart because perceptions of the relative value of certain items changes over time. However, to be able to compare, it is important to include consumptions questions measuring similar domains of poverty in each of the surveys. We therefore restricted the consumption questions included in the survey to similar questions asked between the two surveys and used Principal Components Analysis to construct the asset index. A limitation of the study is certainly that the understanding of whether a household is poor or wealthy is based on six consumption items (1) the presence of clay floors, (2) wood as fuel for cooking, (3) ownership of a stereo system, (4) television, (5) motorbike, or (6) car. In developing this asset index, we do include factors related household consumer haviour as in the stereo system, television, motorbike and car. As well as questions signalling longer term savings/ investments such as the type of floor of the house and the type of fuel used in cooking. So, in essence, we only used the six variables that were comparable between the two surveys because we were constrained by data availability.

We have added a discussion of this to the limitations section of the manuscript: “A limitation of our study includes the measure of household socio-economic position used. Our measure of household level socio-economic position was primarily based on consumption data collected during the prevalence survey and some of the important factors predicting poverty in Việt Nam such as education were not included in this measure. Furthermore, consumption data are sensitive to change over time, for example an item that was a signal of prosperity in 2007 may no longer be a good indicator of wealth in 2017. This limitation was mitigated to some extent by using different household- and neighbourhood level measures of poverty including rural residences, the region where the district is situated with Việt Nam as well as the percentage of people in the district who are considered poor (the district poverty rate). We furthermore did not only rely on results based on the consumption-based asset index, we also estimated the absolute wealth of households and the primary results of the study held across the different measurements used. A further limitation of the study is that while tuberculosis prevalence surveys are valuable for understanding socio-economic differences in tuberculosis prevalence in countries, given that tuberculosis is a relatively rare disease in the population studied, there is limited power to explore the socio-economic drivers of tuberculosis prevalence [14]. However, combining repeated cross-sectional surveys with neighbourhood deprivation estimates during a period of remarkable economic growth provides valuable insights into the dynamics of the relationships between tuberculosis and economic development in Việt Nam”.

45. Line 23: TB is not associated only with the crowding places. Rewrite this statement and insert a reference.

We have updated this paragraph: “Equitable economic development improves neighbourhood economy which improves living conditions through reduced crowding, availability of windows to improve air circulation and reduced periods of transmission in neighbourhoods [31]. Furthermore, improvements in the neighbourhood economy will increase household resources, reducing malnutrition and improving households’ ability to seek healthcare [15]. Comparatively wealthier households will have greater ability to negotiate access to neighbourhood resources such as housing and health services therefore lowering their risk of tuberculosis. If symptomatic (clinical tuberculosis), individuals would be more likely to seek and receive tuberculosis care, reducing transmission periods.”

# **Results**

46. The terms gender and sex are not interchangeable (as discussed in [http://www.who.int/gender/whatisgender/en/](http://protect-eu.mimecast.com/s/OLz0Cl714TzgOryhNfOgO?domain=who.int) ); please use the appropriate term.

The survey collected information on self-reported gender rather than biological sex. We have therefore amended the terms used in the manuscript and are now consistently using the term gender throughout.

Including:

- Table 2, page 15
- Table 1, page 12

47. The results section is well reported. There are major revisions in this section.
    The results reported in this section are not clear. I suggest rearranging this section to make it clearer and more readable according to the STROBE Statement.  
    I suggest to the authors to use the comma to separate the number every third digit from the right.

The results section has now been rewritten for clarity. The completed STROBE checklist for the reporting of cross-sectional studies is attached as supplementary 2.

48. line 15 Please explain what an "illness concentration curve" is.

An illness concentration curve is used to quantify whether inequality in household socio-economic position (SEP) exist for a health sector variable (here, TB prevalence). The curve is a plot of the cumulative proportion of ill-health against the cumulative proportion of the population ranked by SEP, beginning with the least advantaged. We quantify the position of the geometric mean on the curve by estimating the concentration index which is twice the area between the concentration curve and the line of equality which is the 45-degree line on the graph.

49. p. 11 line 17-19 The two measures are also not inconsistent. But these results are inconsistent with the GINI results cited earlier.
    Table 2 Do not categorize age or SES. See my blog post [https://medium.com/@peterflom/what-happens-when-we-categorize-an-independent-variable-in-regression-77d4c5862b6c](https://protect-eu.mimecast.com/s/A3G9CzXkjimJ813cyOfxv?domain=medium.com) 
    The AIC is of no use here. It is only useful for comparing different models on the same data set.

We present age and socio-economic status as categorized in Table 2, however in the regression analyses, we use the age variables as continuous variables.

# **Figures and tables**

50. Please include the total N at the top of table 2 & 3

Now included.

51. Please define abbreviations such in the table footnotes. For example, SEP, AWE, CI

Each of the table footnotes have been amended.

Table 1: “SD: standard deviation. n: sample size. N: population size.”

For Table 2: “Prevalence Ratios (PRs) and Confidence Intervals (CIs) are estimated using log-binomial mixed effects statistical models. Coefficients are weighted for stratification (differential cluster size, participation by age and gender, stratification by areas and post stratification weight). TB: tuberculosis. PR: prevalence ratios. CI: confidence intervals. Ref: reference values. “

For Table 3: “The dataset includes a total of 155,919 participants. Prevalence ratios (PR) were estimated using multi-level mixed effects models with random intercepts. Model A shows individual-level regressors only, Model B shows individual-level and household level variables while using the absolute wealth estimate to understand the impact of household wealth, while Model C uses a relative measure of household socio-economic position (SEP). Provincial poverty rate: the percentage of the population living below US$2 per day. AWE: absolute wealth estimate for the household. CI: Confidence Interval. PR: Prevalence Ratios. SEP: socioeconomic position. Ref: reference values.”

52. Please provide a meaning for the bars and whiskers in figures 2 and 5.

The figure legends have been amended in the following way: “Figure 2. The distribution of tuberculosis prevalence by socioeconomic position (SEP) as measured in the 2007 and 2017 tuberculosis prevalence surveys. The plot shows the average household SEP index (represented by the dots) and the confidence intervals around the mean (the bars around the dot).”

“Figure 5. Illness concentration indices (ConcIn) for 2007 and 2017 Viet Nam TB prevalence surveys. Sampling weights were applied. A negative concentration index means that the health outcome (tuberculosis illness) is concentrated in the poor, while a positive index value means that the disease is concentrated in those who are wealthier. The concentration index (Figure 4) is an expression of the area between the concentration curve (Figure 3) and the line of perfect equality. In the figure, the bars represent the mean with the whiskers representing the distribution of data around the mean. The outlying data points are shown in the circles above and below the whiskers.”

# **Discussion**

53. In the Discussion, please include the limitations of the study, implications and next steps for research, clinical practice, and/or public policy.

We have expanded the conclusion and discussion sections to explain the limitations and policy implications of the study. Specifically, we have added the following section related to the limitations of the study in the discussion:

“A limitation of our study includes the measure of household socio-economic position used which may have led to the misclassification of household socio-economic position given a limited set of consumption data. Our measure of household level socio-economic position was primarily based on consumption data collected during the prevalence survey and some of the important factors predicting poverty in Việt Nam such as education were not included in this measure. Furthermore, consumption data are sensitive to change over time, for example an item that was a signal of prosperity in 2007 may no longer be a good indicator of wealth in 2017. This limitation was mitigated to some extent by using different household- and neighbourhood level measures of poverty including rural residences, the region where the district is situated with Việt Nam as well as the percentage of people in the district who are considered poor (the district poverty rate). We furthermore did not only rely on results based on the consumption-based asset index, but we also estimated the absolute wealth of households and the primary results of the study held across the different measurements used. A further limitation of the study is that while tuberculosis prevalence surveys are valuable for understanding socio-economic differences in tuberculosis prevalence in countries, given that tuberculosis is a relatively rare disease in the population studied, there is limited power to explore the socio-economic drivers of tuberculosis prevalence [14]. However, combining repeated cross-sectional surveys with neighbourhood deprivation estimates during a period of remarkable economic growth provides valuable insights into the dynamics of the relationships between tuberculosis and economic development in Việt Nam. Lastly, it possible that there may have been selection bias due to non-participation in the sampling of respondents during the second survey, in that with improve economic welfare there is less incentive for participants to attend the free screening service provided by the survey with therefore a bias towards poorer households enrolling. Our findings may therefore be underestimating the true population level shift in the tuberculosis burden towards wealthier households.”

Related to the next steps for public policy: “Our results show the potential to tuberculosis prevalence reductions of general and equitable improvements in socioeconomic circumstances in a population. While economic growth, and ensuring resource distribution is equitable, falls outside the remit of specific national tuberculosis programmes, our study strengthens the case for a multi-sectoral response to tuberculosis which we hope gives further encouragement to policies that aim to achieve this.”

54. The discussion section is well reported. There are major revisions in this section.
    I suggest to the authors a discussion based in the data reported in the results section and, how the findings can subsidize the policy makers in different scenarios to obtain the best outcomes in decrease TB burden.  
    I suggest to the authors to insert the limitations of the study in the last paragraphs in the discussion section.  
    Please, discuss the data from survey one and two. Is there a point of integration or similarity?

The discussion section has been expanded as suggested, changes are discussed in more detail in point 53..

55. Editorial changes

- Line 10: Insert a point after the letter "l" in the "at al."
- Line 11 - 13: Please include a reference in this statement. 
  Line 17: Insert a point after the letter "l" in the "at al."
  Line 18: Reference correctly the citation "Carter at al.".
- Page 17, ln 4: I think it might be better to also include 2017 here, because this statement also apply to 2017. In the next sentence you can then clarify that there was a decrease in 2017.
- Page 17, ln 6: Can we add "Increased", so that it read as "associated with increased TB prevalence"
- Page 17, ln 33: Is there any study that quantify the significance of improvement in TB diagnostics? If you have any, please add it.
- Line 35 - 36: Please include a reference in this statement.

Amended.

# **Conclusions**

56. Please include in the conclusion section how this study will support, for example, policy makers to better understanding the impact of socio-economic factors on increase the burden of tuberculosis orientating the efforts to accomplish the aims of The End TB strategy.

We have added additional sentences to make this clearer. “To our knowledge, this is the first study to use repeat direct measurements of tuberculosis burden to empirically examine the relationship between equitable economic development and a reduction in tuberculosis prevalence. We found that with equitable economic growth and a reduction in tuberculosis burden, tuberculosis became less concentrated among the poor in Việt Nam. The study highlights the important contribution of shared resources on not only poverty reduction but also a shift away from differentially impacting on the poorest households.”

# **References**

57. Please ensure in-text reference call outs are enclosed in square brackets. For example, “... countries [1,2]."
58. In the text, cite the reference number in square brackets according to the PLOS Medicine's submission guidelines.

The citation style for all references have now been updated and uses the square brackets.

59. Please include access dates for all weblinks (e.g., Ref #14, 26), and ensure that all weblinks are current and accessible.

Included.

60. Please update ref #23 or delete if not published.

This reference has been updated once published.

61. I suggest to the authors to insert some other references to be included in the discussion section. These studies were carried out in different scenarios and could improve this study.
    [https://doi.org/10.1371/journal.pone.0249822](https://protect-eu.mimecast.com/s/VvG3CqZ14t7mLQDFg7Wyk?domain=doi.org)
    [https://doi.org/10.5588/ijtld.18.0149](https://protect-eu.mimecast.com/s/h11ECr914SDLwqpi5I3HR?domain=doi.org)
    [http://dx.doi.org/10.2471/BLT.06.038331](http://protect-eu.mimecast.com/s/LEGeCvZ14tyMO9GTP0fFy?domain=dx.doi.org)
    [https://doi.org/10.1016/j.jiph.2020.03.010](https://protect-eu.mimecast.com/s/dj40Cwr14SRY0j7szkgS4?domain=doi.org)
    [https://doi.org/10.1016/S1473-3099(09)70041-6](https://protect-eu.mimecast.com/s/-SijCx1g4cQrmlyhmGtyd?domain=doi.org)
    [https://doi.org/10.1111/tmi.13409](https://protect-eu.mimecast.com/s/H7RTCyXjgiy961gTLQKLi?domain=doi.org)

Thank you for providing additional helpful references.
