# Supplementary material for: Social determinants of the changing tuberculosis prevalence in Việt Nam: Analysis of population-level cross-sectional studies
Source: PLoS Med. 2022 Mar 18;19(3):e1003935. doi: 10.1371/journal.pmed.1003935 (PMC8932606; doi:10.1371/journal.pmed.1003935)
Supplement: Attachment — Submitted filename: response.docx [file pmed.1003935.s005.docx]

**Response to reviewer comments**

for manuscript PMEDICINE-D-21-03248R2 “*Social determinants of the changing tuberculosis prevalence in Viet Nam: analysis of population-level cross-sectional studies*”

Thank you for the favourable review of our manuscript, the changes suggested are listed below with a response provided point-by-point.

| *From the Editors* |  |
| --- | --- |
| Thank you for providing a link to the description of the dataset; however, this link is inactive and DOI cannot be found. | This has now been corrected, and the link should be active shortly. |
| Please situate the Author Summary after the Abstract. | Amended as suggested |
| In the last sentence of the Abstract’s Methods and Findings section, please describe the main limitation(s) of the study's methodology. | Added the following sentence(s): “A limitation of our study is that while tuberculosis prevalence surveys are valuable for understanding socioeconomic differences in tuberculosis prevalence in countries, given that tuberculosis is a relatively rare disease in the population studied, there is limited power to explore socioeconomic drivers. However, combining repeated cross-sectional surveys with neighbourhood deprivation estimates during a period of remarkable economic growth provides valuable insights into the dynamics of the relationships between tuberculosis and economic development in Việt Nam.” |
| Thank you for providing your STROBE checklist. Please replace the page numbers with paragraph numbers per section (e.g. "Methods, paragraph 1"), since the page numbers of the final published paper may be different from the page numbers in the current manuscript. | Updated. |
| References: Please include access dates for all weblinks (e.g., Ref #32, and ensure that all weblinks are current and accessible. Please reformat the citation style into PLOS Medicine's format and ensure journal name abbreviations consistently match those found in the National Center for Biotechnology Information (NCBI) databases | Updated, all weblinks checked and last accessed date added. |
| *From Reviewer 1* |  |
| Dear authors, Thank you for revising the manuscript considering the suggestions. The manuscript is now technically sound.  I do not have additional comments on the revision in this manuscript version. | Thank you. |
| *From Reviewer 4* |  |
| The authors have addressed my concerns and I now recoommend publication. Peter Flom | Thank you. |
